# DeepTCR is a deep learning framework for revealing sequence concepts within T-cell repertoires

**DOI:** 10.1038/s41467-021-21879-w

**Published:** 2021-03-11

**Authors:** John-William Sidhom, H. Benjamin Larman, Drew M. Pardoll, Alexander S. Baras

**Affiliations:** 1 Bloomberg Kimmel Institute for Cancer Immunotherapy, Johns Hopkins University School of Medicine, Baltimore, MD USA; 2 The Sidney Kimmel Comprehensive Cancer Center, Johns Hopkins University School of Medicine, Baltimore, MD USA; 3 Department of Biomedical Engineering, Johns Hopkins University School of Medicine, Baltimore, MD USA; 4 Department of Pathology, Johns Hopkins University School of Medicine, Baltimore, MD USA

**Keywords:** Computational models, Functional clustering, Machine learning, Adaptive immunity, Immunogenetics

## Abstract

Deep learning algorithms have been utilized to achieve enhanced performance in pattern-recognition tasks. The ability to learn complex patterns in data has tremendous implications in immunogenomics. T-cell receptor (TCR) sequencing assesses the diversity of the adaptive immune system and allows for modeling its sequence determinants of antigenicity. We present DeepTCR, a suite of unsupervised and supervised deep learning methods able to model highly complex TCR sequencing data by learning a joint representation of a TCR by its CDR3 sequences and V/D/J gene usage. We demonstrate the utility of deep learning to provide an improved ‘featurization’ of the TCR across multiple human and murine datasets, including improved classification of antigen-specific TCRs and extraction of antigen-specific TCRs from noisy single-cell RNA-Seq and T-cell culture-based assays. Our results highlight the flexibility and capacity for deep neural networks to extract meaningful information from complex immunogenomic data for both descriptive and predictive purposes.

## Abstract (web summary)

The advent of high-throughput T-cell receptor sequencing has allowed for the rapid and thorough characterization of the adaptive immune response. Here the authors show how deep learning can reveal both descriptive and predictive sequence concepts within the immune repertoire.

## Introduction

Next-generation sequencing (NGS) has allowed a comprehensive description and understanding of the complexity encoded at the genomic level in a wide variety of organisms. The applications of NGS have grown rapidly as this technology has become a molecular microscope for understanding the genomic basis for the fundamental functions of the cell^1^. In parallel to this explosion of NGS applications, in the machine learning world, deep learning has seen a similar expansion of applications as computational resources have grown; there exist many opportunities to apply deep learning in genomics as the data generated from NGS are very large and highly complex^2–7^.

T cell receptor sequencing (TCR-Seq) is an application of NGS that has allowed scientists across many disciplines to characterize the diversity of the adaptive immune response^8–18^. By selectively amplifying and sequencing the highly diverse antigen-specific CDR3 region of the β-chain of the T cell receptor, scientists have been able to study clonal expansion as a probe for responses to both foreign and native potential antigens^19^. With this new sequencing technology, there has arisen a need to develop analytical tools to parse and draw meaningful concepts from the data (such as those pertaining to shared sequence concepts or motifs), since antigen-specific T cells exist within a sea of T cells with specificities irrelevant to the microbe or tumor cell being assessed. In recent work, investigators have applied conventional sequence analytics, where either targeted motif searches or sequence alignment algorithms have been used to begin parsing the data within TCR-Seq^20–22^. However, identifying signal over noise is particularly challenging in studying in vivo T cell responses such as tumor-specific T cell responses, which are mediated by a small proportion of the overall pool of tumor-infiltrating lymphocytes and peripheral blood lymphocytes^23–25^. While these CDR3 alignment algorithms have been used successfully to assign TCRs to a limited number of antigens after multimer sorting, they have done so in absence of the 100–1000× background of irrelevant specificities seen in typical in vivo T cell responses^21,22^.

## Results

### Deep learning approach

In light of this need to better featurize TCR sequences, we turned to deep learning primarily through the use of convolutional neural networks (CNNs) as a powerful means to extract important features from sequencing data for both descriptive and predictive purposes. As has been demonstrated in previous genomic applications of deep learning, the main advantage of CNNs in this application is the ability to learn these sequence motifs (referred to as kernels in this context) through some objective function given to the network^4^. These learned motifs can then be used as part of a complex deep learning model to either describe the data in a new latent space or be used for a classification task. Furthermore, since the initial conception and presentation of this work, multiple groups have begun to recognize the value of deep learning and broader machine learning techniques in this endeavor to learn these sequence concepts of immune receptors^26–30^.

We present DeepTCR, a platform of both unsupervised and supervised deep learning that is able to be applied at the level of individual T cell receptor sequences as well as at the level of whole T cell repertoires, which can learn patterns in the data that may be used for both descriptive and predictive purposes. In order to demonstrate the utility of these algorithms, we collected a variety of TCR-Seq datasets including samples sorted by antigen specificity^20–22^, samples collected from single-cell RNA-seq experiments (10x_Genomics), and samples collected from a novel experimental assay used in detecting functional expansion of T cells^31^ (full dataset details in Supplementary Fig. 1). Across these various datasets, the level of non-specific signal varies given the technical difficulties associated with extracting true non-specific signatures of TCR responses, and we seek to demonstrate the value of applying deep learning in these scenarios to leverage knowledge about sequence homology to extract the true antigen-specific signals.

### TCR featurization

**Fig. 1:**
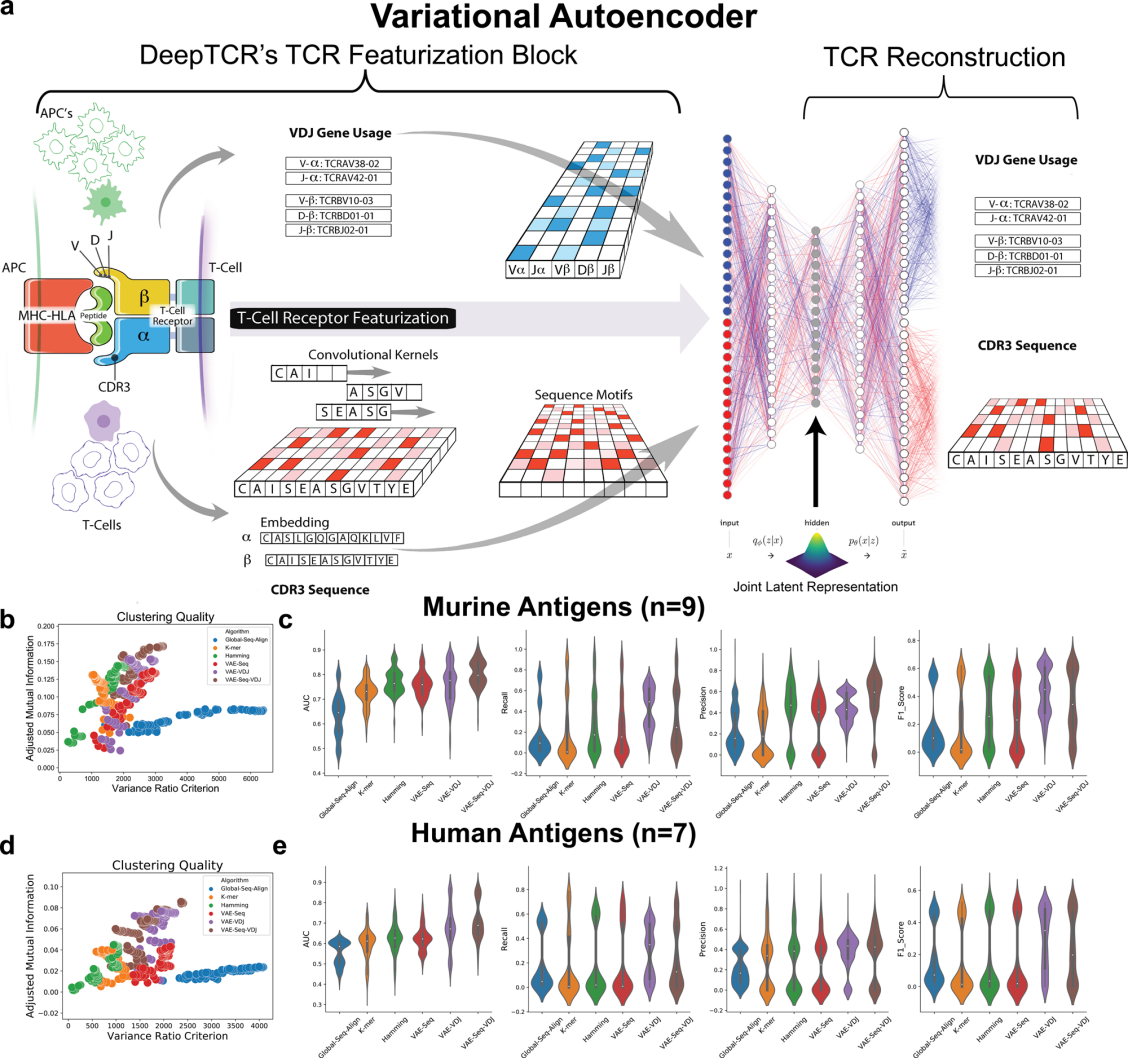
Unsupervised TCR sequence representation. **a** In order to represent a T cell receptor (TCR) we have implemented a variational autoencoder (VAE) to take the CDR3 sequences from both the α- and β-chains along with their corresponding V, D, and J gene usage and learn a joint representation of these inputs. The CDR3 sequences serve as input to the encoder side of the network where convolutional layers are then applied to learn sequence motifs from these regions. As for the V/D/J gene inputs, these are represented as categorical variables which are then transformed with a trainable embedding layer to learn a continuous representation of the gene usage of a given TCR. Finally, these inputs are concatenated together passed through fully connected layers to result in the latent representation of the TCR. This latent representation is then sampled from in order to reconstruct the input CDR3 sequences and V/D/J genes through the decoder side of the network. Finally, the weights of the neural network are trained via gradient descent to jointly minimize both the reconstruction and variational loss. The trained network is then used to take a given TCR and represent it in a continuous numerical domain for downstream analysis such as clustering. **b**, **d** In order to assess the quality of various methods of TCR featurization, we derived TCR distances from the various featurization methods (VAE-Seq, VAE-VDJ, VAE-Seq-VDJ, Hamming, K-mer, Global-Seq-Align) and applied an agglomerative clustering algorithm varying the number of clusters evenly from 5 to 100 and measured the variance ratio criterion of the clustering solutions and the adjusted mutual information from the clustering solutions to the ground truth antigen labels for both nine murine and seven human antigens. Featurization methods that encourage high-quality clusters that capture a high degree of information of the label (i.e. antigen specificity) should have a high variance ratio criterion and high adjusted mutual information. **c**, **e** In order to benchmark the ability of various methods of TCR featurization to correctly classify a TCR sequence to its antigen, we applied a *K*-Nearest-Neighbors instance-based classification algorithm (varying *K* evenly from 1 to 500, Supplementary Figs. 3–10) to the derived TCR distances on the nine murine and seven human tetramer-sorted antigen-specific T cells and assessed classification performance via fivefold cross-validation strategy, measuring AUC, Recall, Precision, and *F*1 Score. Illustrations for Panel **a** provided by Tim Phelps Copyright 2020 JHU AAM, Department of Art as Applied to Medicine, The Johns Hopkins University School of Medicine.

The main building block of all architectures in DeepTCR utilizes a common method of TCR featurization (Fig. 1a). First, any of the available α- or β-chain CDR3 variable length sequences are provided to the network and are embedded via the use of a trainable embedding layer, as described by Sidhom et al.^7^, to learn properties/features of the amino acids and transform the sequences from a discrete to continuous numerical space. Subsequently, a three-layer CNN is used to extract sequence-based features from both chains. Additionally, V/D/J gene usage is provided to the network as a categorical variable in a “one-hot” representation. A trainable embedding layer is again leveraged to learn features of the V/D/J gene segments and transform them from a discrete to continuous numerical space. These features are then concatenated within the network to provide a joint representation of the TCR sequence through its CDR3 sequences and V/D/J gene usage, allowing a more complete representation of the T cell receptor.

### A variational autoencoder provides superior antigen-specific clustering

We first implemented this method of TCR featurization within the unsupervised learning setting in order to learn the underlying distribution of the sequence data in high-dimensional space for the purpose of clustering TCR sequences that likely recognize the same antigen as this is a commonly performed analysis in TCR-Seq. In order to learn the underlying distribution of the data in a latent space informed by our representation of TCRs, we implemented a variational autoencoder (VAE) as autoencoders have been previously described as a common dimensionality reduction/data re-representation technique^32,33^. Our implementation of a VAE (Fig. 1a) starts by taking a TCR CDR3 sequence and corresponding V/D/J gene usage and following featurization as described previously, is transformed into a latent space that is parametrized by a multidimensional unit Gaussian distribution. The sequences and V/D/J gene inputs are then reconstructed from the latent space through the use of deconvolutional and fully connected layers. When the network is trained, one can extract the latent features that represent information from both the CDR3 sequences as well as the V/D/J gene usage in a format that is conducive to downstream analyses such as clustering.

In order to initially assess the value of using deep learning as method of TCR featurization, we collected data for tetramer-sorted antigen-specific cells for nine murine (Db-F2, Db-M45, Db-NP, Db-PA, Db-PB1, Kb-M38, Kb-SIY, Kb-TRP2, Kb-m139) and seven human (A1-CTELKLSDY, A1-VTEHDTLLY, A2-GILGFVFTL, A2-GLCTLVAML, A2-NLVPMVATV, B7-LPRRSGAAGA, B7-TPRVTGGGAM) antigens where the ground truth label corresponds to a particular antigen specificity for an individual sequence^20–22^. We benchmarked the VAE against featurizations of TCRs based on Hamming distances, K-mer representation, and global sequence alignment. Prior methods including GLIPH and TCRdist both use Hamming distances while ImmunoMap uses a global sequence alignment^20–22^. Our implementation of the Hamming distance method was directly benchmarked on the Glanville_2017 dataset against the original GLIPH algorithm demonstrating improved clustering accuracy as measured in the original manuscript (Supplementary Fig. 2). For the VAE, we benchmarked the algorithm with different types of inputs to the network, including just the β-chain CDR3 (VAE-Seq), just the V/D/J gene usage (VAE-VDJ), and the combination of both inputs (VAE-Seq-VDJ). First, to benchmark these various methods of featurization in clustering antigen-specific TCRs, we ran an agglomerative clustering algorithm varying the number of clusters from 5 to 100 and then assessed the variance ratio criterion of the clustering solutions and the adjusted mutual information from the clustering solutions to the ground truth antigen labels (scikit-learn)^34,35^. We noted that the VAE methods maintained the highest variance ratio criterion while also maintaining a high adjusted mutual information to the ground truth labels for both murine and human datasets (Fig. 1b, d) suggesting VAE-based methods form high-quality clusters that correspond to the true antigen-specific labels. To further query the value of these learned features in correctly clustering sequences of the same specificity, we applied a *K*-Nearest Neighbors Algorithm across a wide range of *K* values using a fivefold cross-validation strategy and assessed performance metrics of the classifier including AUC, Recall, Precision, and *F*1 Score for all featurization methods (Fig. 1c, e and Supplementary Figs. 3–10)^36^. We noted that across all performance metrics, the VAE-based methods (at least one) outperformed current state-of-the-art approaches for TCR featurization. Furthermore, using both sequence and V/D/J gene usage resulted in the highest AUC performance for both the murine and human antigens, suggesting both types of inputs provide distinct and contributary information to antigen specificity assignment in addition to encouraging a featurization of the TCR that is length invariant (Supplementary Fig. 11). These results demonstrate that a deep learning approach able to incorporate both sequence-level information and V/D/J gene usage results in optimal antigen-specific clustering of TCR sequences as compared to current state-of-the-art methods.

### Supervised approaches improve antigen-specific classification

**Fig. 2:**
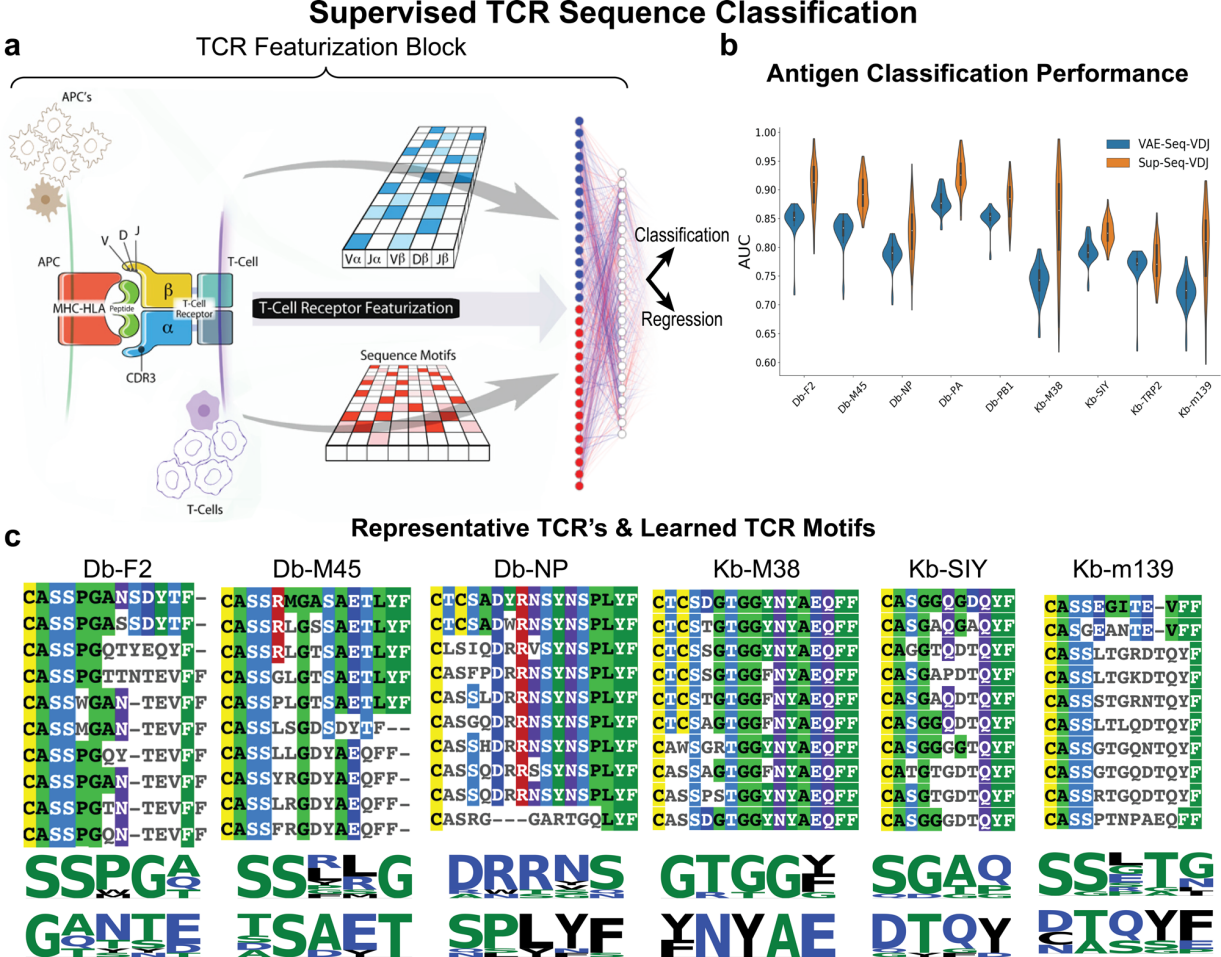
Supervised TCR sequence classification. **a** Network architecture schema: Previously described TCR featurization block is implemented to featurize a TCR sequence and then either output a label (i.e. antigen specificity) or continuous regressed value (i.e. affinity measurement). **b** Supervised TCR Sequence classifier was trained/tested on nine murine antigen-specific TCR sequences via a 100-fold Monte-Carlo cross-validation strategy where classification performance, assessed via AUC measurements, was measured on the test sets. Classification performance was benchmarked against DeepTCRs unsupervised VAE instance-based learning classifier as shown here and classical Random Forest (RF) and Support Vector Machine (SVM) algorithms (Supplementary Fig. 12). **c** Representative Db and Kb murine antigens where top predicted CDR3 sequences are shown via multiple-sequence alignment and learned kernels for these representative sequences are visualized below the alignment. Illustrations for Panel **a** provided by Tim Phelps Copyright 2020 JHU AAM, Department of Art as Applied to Medicine, The Johns Hopkins University School of Medicine.

While our unsupervised VAE approach demonstrated superior performance to state-of-the-art methods through utilizing a joint representation of the TCR from its V/D/J gene usage and CDR3 sequence, we wanted to see if using a supervised machine learning approach could improve performance even more (on the same dataset as previously described for the unsupervised classification task). To do so, we developed a fully supervised model that learns sequence-specific motifs to correctly classify sequences by their antigen-specific labels (Fig. 2a), in which we observed that our supervised approach improved performance over the previously described unsupervised VAE approach (Fig. 2b) and a more conventional Random Forest (RF) & Support Vector Machine (SVM) (Supplementary Fig. 12)^37,38^. In addition, being able to extract knowledge from the network can inform relevant motifs for antigen-specific recognition. Therefore, we established a method by which we could identify the most predictive (i.e. representative) sequences for a given class and query the associated learned kernels/motifs (Fig. 2c). Following training, we were able to sort all the sequences by the predicted values of the network to identify the sequences most predicted to bind a given antigen, and then in order to identify the associated motifs to those sequences, we assessed the association of the learned motifs by the network to these prediction values via multinomial linear regression where the β-coefficients of the linear model correspond to the level of association between a given kernel/motif and the predicted probability of a TCR being antigen specific. Motifs that were highly associated with the predicted probability of binding a given antigen were displayed with Logomaker^39^.

### Supervised regression allows identification of antigen-specific TCRs in single-cell data

**Fig. 3:**
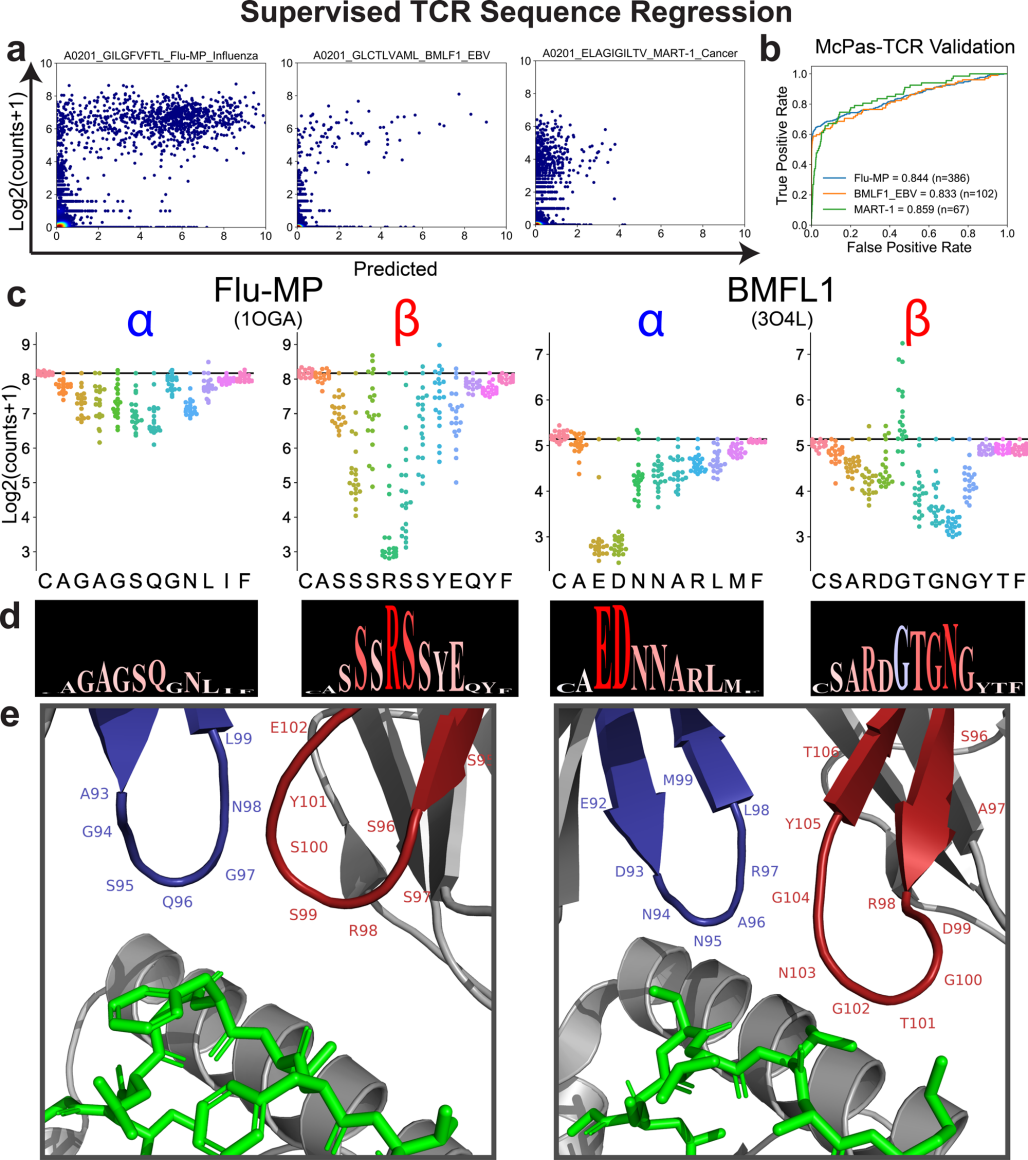
Supervised TCR sequence regression. **a** In order to test the ability of a supervised deep learning method to learn and regress continuous value outputs, we collected published single-cell data from 10x Genomics where 57,229 unique α/β pairs were collected with a count-based measurement (as a proxy for binding affinity) to 44 specific peptide-MHC (pMHC) multimers and 6 negative controls. A fivefold cross-validation strategy was employed on every antigen to obtain independently predicted regression values for every α/β pair to a given antigen and predicted vs actual counts are shown for a select three antigens. **b** For the shown epitopes, experimentally derived antigen-specific CDR3 β TCR sequences were collected from the McPAS-TCR database and models trained on the 10x Genomics dataset were applied to this independent dataset of TCRs to assess the classification performance via examining the ROC curves and their corresponding AUCs. **c** For the Flu-MP and BMLF1 epitopes where data from the 10x Genomics dataset were available to train our models, crystal structures and their corresponding TCR CDR3 sequences were also collected from The Protein Data Bank and permutation analysis was conducted to analyze the sensitivity of each residue to the predicted binding affinity from our deep learning model. The results of this model are shown for the corresponding α- and β-chain for both antigens. **d** To create compact representations of the information in our residue sensitivity analysis, we propose a visualization of this information termed a Residue Sensitivity Logo (RSL). **e** Crystal structures highlighting the relevant α (blue) and β (red) CDR3 regions. Predictive performance of Residue Sensitivity analysis to identify known contact residues shown in Supplementary Fig. 13.

The binding of a TCR to a peptide-major histocompatibility complex (pMHC) is not usually considered a binary phenomenon but rather one that is characterized by a binding affinity. Therefore, we proposed to use DeepTCR to regress UMI (unique molecular identifier) counts as a proxy for binding affinity (a caveat to this assumption being that differing TCR expression levels can also affect the UMI counts) as available in a second single-cell dataset published by 10x Genomics where the binding to cognate T cells of 57,229 unique α/β pairs to 44 specific pMHC multimers and 6 negative controls was characterized. DeepTCR was able to identify TCRs that have both high observed UMI counts and a predictive signature, providing a tool to better isolate antigen-specific TCRs (Fig. 3a). To independently validate whether these models trained on single-cell data learned salient antigen-specific features of the immune response, we collected experimentally validated CDR3 β sequences from the McPAS-TCR database^40^ for Flu-MP (influenza derived), BMLF1 (EBV derived), and MART1 (melanoma derived) epitopes and applied the respective models trained on the 10x Genomics dataset on these TCRs. We specifically removed any TCR sequences from this independent validation cohort that were in the data used to train the models. We observed that these models were able to predict antigen-specific TCRs in the independent and experimentally validated dataset from the McPAS-TCR database with a high level of accuracy (Fig. 3b), which suggests that despite the inherent noise of a tetramer-based assay on which the models were trained, our algorithm could extract the salient antigen-specific features of the TCR.

### Perturbation analysis reveals important residues

One of the advantages of training a predictive model is the ability to perturb its inputs and measure the change in the output of the model; in other words, being able to conduct a sensitivity analysis. Assuming the model has correctly learned the rules of antigen specificity, one can identify residues in a TCR sequence that are highly sensitive to change in a causal fashion and thus describe the relative importance of any given residue to antigen-specific binding. In order to demonstrate the utility of such an approach, we collected crystallography data from The Protein Data Bank for Flu-MP (1OGA) and BMLF1 (3O4L), two antigens for which we had trained a model to predict binding affinity from the 10x Genomics dataset^41–43^. We first took the sequence data from the CDR3 regions of these TCRs and permuted each position of each sequence with all other 19 amino acid and obtained predicted affinities for each single amino acid mutation in order to see which residues were sensitive to change (Fig. 3c). We first noted that certain positions were highly sensitive to any change in amino acid (i.e. R at β-6 in Flu-MP), suggesting the importance of that particular residue for binding in the context of that TCR. We also observed that we could assess the contribution of the α- vs β-chain to specificity with this analysis by comparing the general sensitivity to perturbation between the two chains. For example, we noted that the Flu-MP TCR is more sensitive to perturbation in the β-chain whereas the BMLF1 epitope shows similar sensitivity to perturbations in either the α- or β-chain. Finally, while most perturbations lowered the predictive binding affinity of the given TCR to its cognate antigen, we noted that for the BMLF1 TCR, the G at β-6 demonstrated that many perturbations at that site would actually increase the binding affinity of this TCR, suggesting that this approach could also be utilized for TCR engineering to design high-affinity TCRs. To represent this information about each residue in a more compact visualization, we created Residue Sensitivity Logos (RSLs) which allow for rapid comparison of many sequences in a logo type format where the size of the residue corresponds to sensitivity at that particular position and the color scheme represents the average direction of changes at that position to the binding affinity (Fig. 3d). For example, the β-6 in Flu-MP is large because it is sensitive to any perturbation and it is colored red because most perturbations at that site would result in a lower binding affinity whereas β-6 in the BMLF1 TCR is also large because it is also sensitive to perturbation but it is colored blue because most perturbations at that site would increase the binding affinity. Finally, we correlated information from these RSLs to the crystal structure (Fig. 3e). By assessing how well the quantified sensitivity at a given position predicted whether that position was a contact residue (AUC: 1OGA = 0.824, 3O4L = 0.907), we demonstrated that the information learned by our neural network was congruent with known important residues in these respective TCRs (Supplementary Fig. 13)^22^. These results suggest that by combining the high-throughput nature of single-cell technology with deep learning as illustrated in these examples, one can obtain a robust understanding of the sequence determinants of TCR antigenicity as well as provide guidance for TCR engineering.

### TCR repertoire classification identifies TCR signatures of an elite suppressor of HIV

**Fig. 4:**
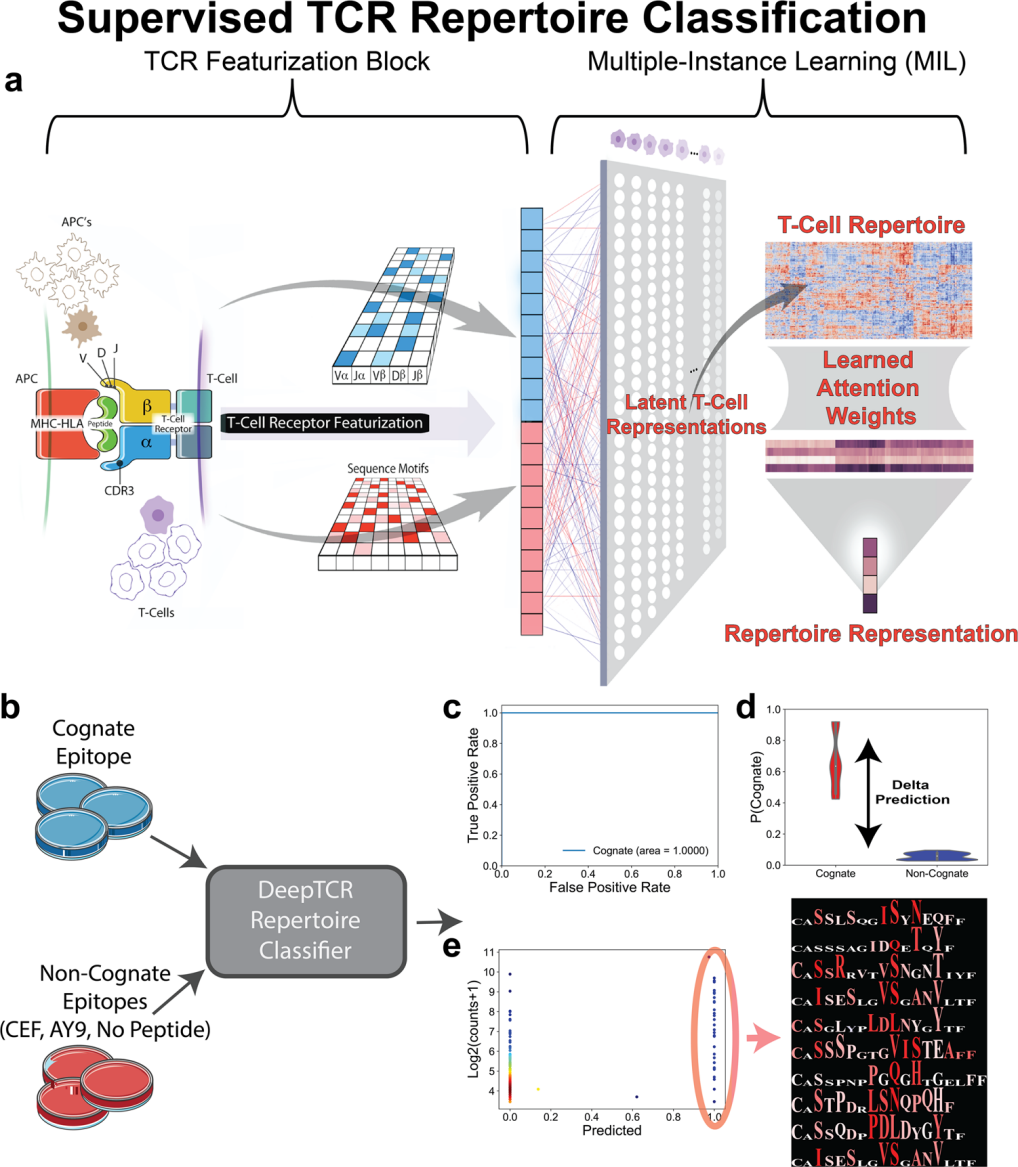
Supervised TCR repertoire classification. **a** Multiple-Instance Learner (MIL) for classifying a TCR repertoire. Following previously described the TCR Featurization Block, we implement a multi-head attention mechanism to make sequence assignments to concepts within the sample. The number of concepts in the model is a hyperparameter, which can be varied by the user depending on the heterogeneity expected in the repertoires. Of note, this assignment of a sequence to a concept is done through an adaptive activation function that outputs a value between 0 and 1, allowing the network to put attention on the sequences that are relevant to the learning task. When taking the average of these assignments over all the cells in a repertoire, this results in a value within the neural network that directly corresponds to the proportion of the repertoire that is described by that learned concept. These proportions of concepts in the repertoire are then sent into a final traditional classification layer. **b** In order to train TCR Repertoire Classifier on T Cell culture data, the model was given triplicates for the cognate epitope as well as non-cognate controls (CEF, AY9, No Peptide conditions). The model is trained to learn the distinguishing TCR sequence features of the cognate epitope from the controls through 100 Monte-Carlo simulations where the model is trained on two out of the three triplicates and performance is assessed on the left-out well for each set of conditions, ensuring that any predictions used for downstream interpretation have been obtained from data not used in training. **c** If the model is able to distinguish the cognate epitope from the controls with a high level of performance assessed by ROC, the epitope is considered to have elicited an antigen-specific response. **d** The effect size of this response is then quantified by the difference in the magnitude of the average predictions for wells in cognate condition vs the average predictions for wells in the non-cognate or control conditions. **e** Following training of the model, sequence-level predictions can be obtained by running each TCR sequence in the cognate wells through the repertoire classifier allowing extraction of the antigen-specific sequences from the background noise of the T cell culture. Residue Sensitivity Logos are shown for select antigen-specific TCRs. Illustrations for panel **a** provided by Tim Phelps Copyright 2020 JHU AAM, Department of Art as Applied to Medicine, The Johns Hopkins University School of Medicine.

Building on the supervised sequence classifier, we then wanted to design an architecture that could learn from a label applied to a whole repertoire of TCR sequences, most of which are irrelevant to the antigen of interest. This type of problem can be poised as “weakly supervised” as the repertoire label may only apply to a subset of the sequences^44^. Our supervised repertoire classifier was formulated as a supervised multi-instance learning algorithm that is able to extract meaningful concepts that may lie within large repertoires of many sequences (Fig. 4a). This scenario is akin to many use-cases of TCR-Seq where ground truth labels (i.e. experimental exposures, therapies, clinical outcomes) apply to an entire repertoire of TCR sequences and not to any individual sequence. To test the utility of this approach, we collected data from published TCR-Seq data of an assay where T cells from an elite suppressor (ES8) of HIV were cultured with autologous HIV-1 Gag and Nef epitope variants and sequenced after culture to determine the immune repertoire against each epitope^31,45–49^. In the original work, TCR sequences were deemed to be antigen specific if they met certain statistical requirements based on the read count of a given sequence, a proxy for clonal expansion. However, given that T cell expansion in culture in the presence of stimulatory cytokines can occur independent of antigen recognition, we wanted to take advantage of deep learning to leverage the TCR sequence and not just the read count in determining whether an epitope elicited an antigen-specific immune response. We hypothesized that if a well had an antigen-specific response, its T cell repertoire should be distinguishable via its sequence concepts from those not specific for the stimulating peptide(s) (CEF, AY9, No Peptide). In contrast to previously described models herein, our model would make a prediction about the entire T cell repertoire in a well and not any individual sequence, as we would not expect the majority of T cells within a well expanding to a given epitope to be antigen specific. Therefore, we trained a repertoire classifier to predict if the well had been treated by the cognate epitope, or non-cognate conditions (CEF, AY9, No Peptide) given its T cell repertoire (Fig. 4b). If a model could distinguish an experimental/cognate well from the controls based on the T cell repertoire, it would be deemed to be antigen specific. A representative positive cognate epitope is shown where the AUC for the cognate epitope in this classification problem is 1.0, suggesting that the repertoire against this epitope is statistically distinguishable from the controls leading us to believe that this is an antigen-specific response (Fig. 4c). Furthermore, we can measure the magnitude of the difference between the cognate epitope repertoire and the controls by measuring the difference between the average predictions for epitope-specific wells vs controls, termed Delta Prediction (Fig. 4d). While we utilize the AUC as a non-parametric rank based statistical test, the difference in average prediction values between the antigen-specific well and controls is a measure of the magnitude of this difference or the effect size. Once the classifier has been trained, single sequence predictions can be obtained by running each sequence separately through the trained model. This allows us to identify the most predictive sequences against a given epitope. As can be seen for the example epitope, the highly predictive sequences represent only a minority of unique TCRs in the antigen-specific wells and are often not the sequences with the highest read counts (Fig. 4e).

**Fig. 5:**
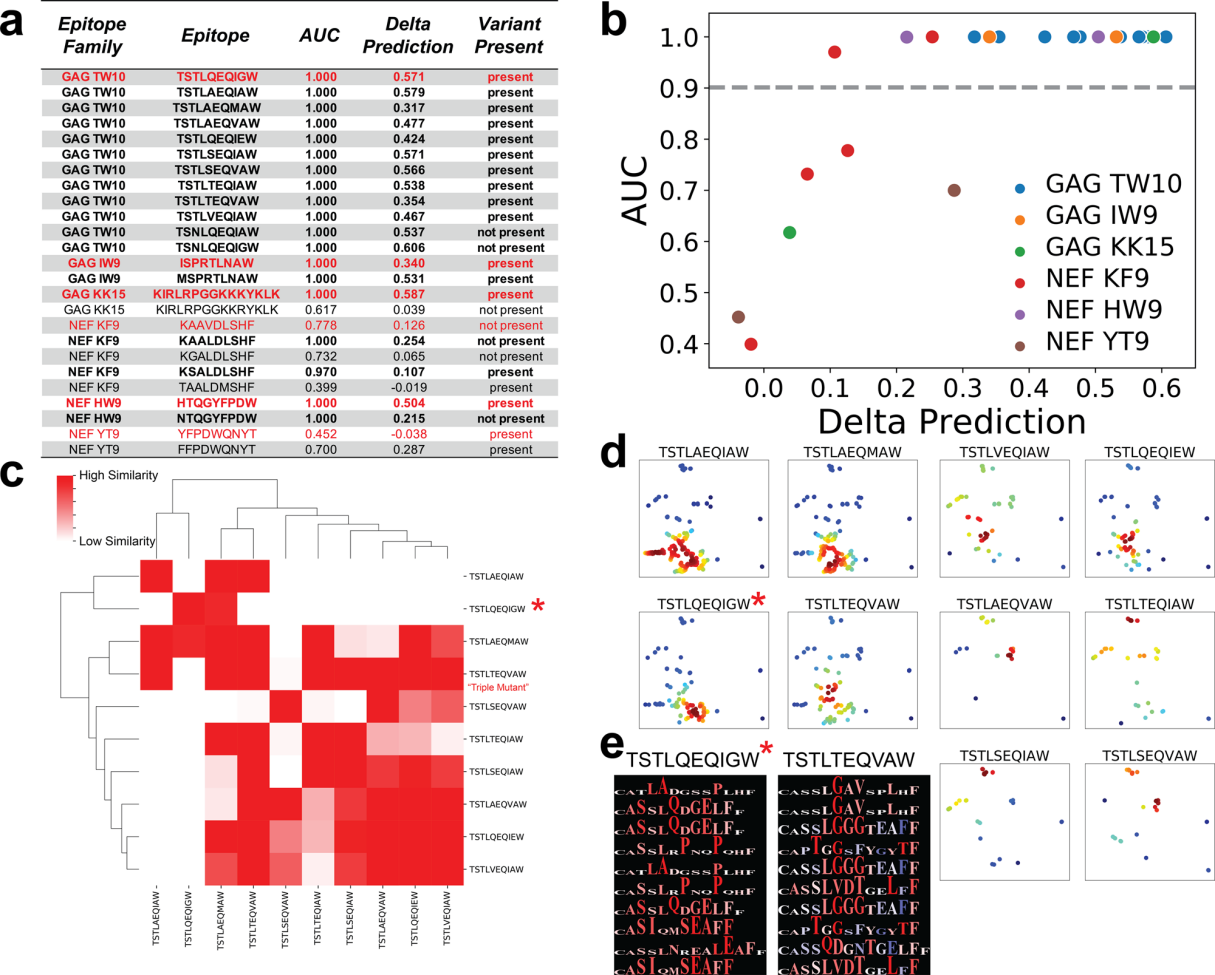
Characterization of TCR repertoire to HIV-specific epitopes. **a** Table summarizing results from all epitopes screened for antigen-specific immune responses via DeepTCR repertoire classifier. Consensus epitopes are denoted in red. Epitopes that were considered to be statistically significant for antigen-specific expansion via AUC > 0.90 are denoted in bold lettering. **b** Delta Predictions vs AUC shown for all epitopes screened for antigen-specific immune responses via DeepTCRs repertoire classifier. **c** All detected variants in ES8 for the GAG TW10 epitope family were collected and DeepTCRs repertoire classifier was ran for all pairwise combinations of these variants. 1-Delta Predictions are plotted in clustered heatmap for simultaneous comparison of immune repertoires where a large value denotes similarity between repertoires. * denotes the consensus sequence. **d** DeepTCR sequence classifier was trained in a multi-class fashion (Supplementary Fig. 15) on the positive predicted sequences (prob > 0.99) from the initial screen against non-cognate epitopes to learn TCR sequence-specific features that could distinguish responses between variants of the GAG TW10 epitope family. UMAP dimensionality reduction was applied to the per-sequence prediction values to generate visualizations for the antigen-specific TCRs. Intensity of coloring corresponds to density as computed by Gaussian kernel density estimation. **e** Residue Sensitivity Logos were produced for the consensus and triple-mutant epitope to highlight structural differences of the immune response.

When we ran this pipeline across the 25 tested epitopes, we noted that our model predicted that 19 of these epitopes (covering 5 out of the 6 epitope families) elicited highly distinguishable sequence features of the T cell repertoire within this elite suppressor when selecting for epitopes that passed a statistical threshold for AUC above 0.90 between the antigen-specific wells and controls (Fig. 5a, b). Furthermore, when applying this trained repertoire classifier for TCR-level inference, we noted that 17/18 (Fisher’s exact test: *p* < 1e−10) of the originally reported experimentally validated TCR-peptide pairs were correctly predicted to be cognate binders (Supplementary Fig. 14)^31^.

Given the breadth of the epitopes this particular elite suppressor responded to, we wanted to characterize the immune repertoire that responded to these epitopes and whether the number and extent of escape variants affected the sequence diversity of the immune repertoire. In order to ask this question, we took epitopes from the epitope families that had at least two autologous variants with detectable immune responses (via previously described method) and conducted all pairwise comparisons of these escape variants within a given epitope family. By training a model for each pair of epitopes within an epitope family, we could measure how distinguishable the repertoire was between any two given variants. A model that could not distinguish between two variants would suggest that the immune repertoire was homologous and thus cross-reactive to both of these variants. On the contrary, if a model could distinguish the immune repertoire between variants, then it would suggest that divergent immune responses were elicited by these variants.

For ES8, the original investigators queried 1 consensus and 11 escape variants of the GAG TW10 epitope family, 10 of which this particular elite suppressor had acquired and as detected via sensitive RT-PCR in either plasma or pro-viral samples, representing the epitope family of highest acquired escape variants. Following training models on all pairwise variants as described previously, a clustered heatmap was used to visualize the pairwise 1-Delta Predictions to compare the immune repertoires of these 10 autologous epitope variants (Fig. 5c) where a large value denotes similarity between the any pair of repertoires. When examining this clustered heatmap, we noted that the consensus epitope (denoted by *) was distinguishable from most the acquired escape variants in ES8. This finding was consistent with the original IFN-γ based work which, for example, demonstrated that the Q244T/I247V/G238A triple mutant could not be recognized by other elite suppressors who had immune responses to the consensus epitope, suggesting that this immune response was novel^46,50^.

We then desired to examine the differences between the immune responses to the GAG TW10 epitope at the individual sequence level so we collected all positive TCRs from our initial screen of these 10 autologous variants and trained a sequence classifier on this “de-noised” data to learn the distinguishing features at the TCR sequence level (Supplementary Fig. 15). Following training this in this multi-class fashion, we extracted the per-sequence predictions across all classes and applied a UMAP dimensionality reduction to visualize the sequences for each variant (Fig. 5d)^51^. Interestingly, when comparing the immune repertoires of the escape variants to the consensus epitope, we noted that while the consensus epitope elicited a relatively focused repertoire, many of the escape variants elicited rather heterogeneous responses based on TCR diversity. Despite many of these escape variants eliciting immune responses, as suggested by our method and validated by previous IFN-γ assays, these variants due to their structural heterogeneity may represent less specific responses. Finally, we wanted to demonstrate the ability of our method to also visualize the differences between any pair of immune responses, so we created RSLs for the consensus epitope (TSTLQEQIGW) and the “triple-mutant” variant (TSTLTEQVAW) demonstrating the differences between these two immune responses (Fig. 5e). These findings lead us to believe that the GAG TW10 epitope is under considerable immune pressure where escape variants often create TCR repertoires that are not only distinguishable from the repertoire against the consensus epitope but also are far more heterogeneous, suggesting less specific immune responses are generated against these escape variants.

In contrast to the GAG TW10 epitope family, the GAG IW9 family had only two variants (the consensus epitope - ISPRTLNAW and the I147M escape variant - MSPRTLNAW) that both generated immune responses. However, these responses were highly homologous given a trained repertoire classifier could not distinguish between wells of either condition, suggesting a cross-reactive repertoire to both these escape variants (Supplementary Fig. 16). This finding was consistent with the initial IFN-*γ* based approaches that found both the consensus and the escape variant both generated immune responses and furthermore, the I127M escape variant was recognized by other subjects who did not have that acquired mutation, confirming our model learned the true cross-reactive nature of this repertoire^46^. These findings suggest that, as has been hypothesized in prior work, certain epitopes may be under stronger immune pressure than others. Our results demonstrate the power of leveraging deep learning on routine T cell culture coupled with TCR sequencing to identify antigen-specific responses that not only can detect the presence of an immune response but also characterize the TCR sequence diversity of that response. However, while our methods cannot prove or make claims around the nature of the immune pressure and its role in HIV pathogenesis, we do demonstrate here the utility for such an approach to generate novel hypotheses that were possibly previously unappreciated.

## Discussion

NGS has become one of the largest sources of big data in the biological sciences, and deep learning is a promising modality for analyzing this kind of big data. In this work, we present DeepTCR, a collection of unsupervised and supervised deep learning approaches to characterize TCR-Seq data for both descriptive and predictive purposes. We first demonstrate that by using a VAE to do unsupervised learning with an improved method of TCR featurization, we can better cluster antigen-specific TCRs. We believe that the real novelty of this approach is allow for joint representations of data inputs of different types (i.e. sequence vs categorical data). While previous methods including GLIPH have used V/D/J information to strengthen the certainty of any given cluster being antigen specific, the initial clustering algorithm does not take into account the V/D/J gene information. In contrast, the TCRdist algorithm does include this information more directly into the computation of its distance metric by incorporating sequence information from the CDR1 and CDR2 regions; the only limitation being this sequence information is most often not collected by current commercial TCR-Seq platforms that often only report the CDR3 sequence and respective V/D/J gene usage. While we only explore featurizing a TCR by its CDR3 sequence and V/D/J gene usage, one can imagine this framework can be expanded to include other information about a TCR such as human leukocyte antigen-context within which it was observed within or even other types of sample-level data such as experimental conditions or treatments a given repertoire was exposed to. Leveraging deep learning allows one the flexibility to generate a rich feature space that can take into account many types of data.

More significantly, we develop supervised methods in applications where labels can greatly help the learning process, such as when there is buried signal in a large sample of sequences. TCR repertoire data are biologically noisy as irrelevant T cells often engage in immune surveillance and can be present without having an antigen-specific role in an immune response. This can make analyses very difficult in the setting of in vivo repertoires where the relevant immune response may only play a small role in the immune response^23^. Furthermore, the most abundant clones within an in vivo response may not necessarily be relevant to any given immune response as immune responses to common viral epitopes can be at high circulating frequencies^52^. Being able to leverage information about the CDR3 sequence can be powerful in extracting signal from the noise not only in in vivo analyses but also in in vitro assays. We demonstrate within this work that when applying deep learning to inherently noisy tetramer-specific or T cell culture expanded clones, we can possibly “de-noise” these assays to isolate true antigen-specific TCRs, showing the ability for deep learning models to extract antigen-specific signal from the background noise of the innate TCR repertoire.

Significant limitations still exist within our analysis and more broadly within the study of immune repertoire. The first being the few and minimally curated datasets that exist at this time. For example, Glanville et al.^22^ and Dash et al.^21^, while publishing high-quality datasets that link TCR to epitope, only assayed a handful of antigens while the immune repertoire has the potential to recognize thousands of antigens with extremely high resolution. While we demonstrate in the HIV dataset that our model can potentially differentiate immune repertoires against epitopes with high homology, datasets do not exist at this time that link TCR to a set of highly homologous epitopes to test our methods against. We imagine in the future, technologies such as the 10x Genomics platform, previously presented in this publication, will help rapidly create the larger datasets needed to better link TCR to epitope. Furthermore, datasets to train and test repertoire classifiers are even more lacking. While the field often assumes that certain exposures or pathology shape the immune repertoire (as in CMV exposed individuals)^14^, the extent of this change is still largely unknown. Arguably, methods such as the ones shown in this paper may be uniquely able to discover these changes for the first time. Additionally, while the data that exist to train these models comes through high-throughput methods such as tetramer sorting^21,22^ or T cell stimulation assays^31^, these methods often introduce some level of noise due to non-specific binding or stimulation. Unfortunately, gold standard methods that would require isolation and cloning of T cell receptors to specifically interrogate the specificity of a given TCR are highly laborious and low throughput. And while findings from our proposed methods would ultimately need to be validated through these more rigorous methods, we do believe that these proposed methods are capable of learning the salient signal from the noise present as is evidenced from the predictive power of these models presented in this work. Finally, Google’s DeepMind recently demonstrated remarkable improvements in performance to predict full three-dimensional (3D) structure from linear sequences of proteins through the use of a deep learning^53,54^. Since T cell receptor function is ultimately tied to its 3D structure (a derivative from the linear sequence) and its interaction with its cognate epitope, it is plausible that our models are capable of learning information about local 3D structure of the T cell receptor.

As sequencing-based technologies only become more ubiquitous, algorithms such as the one presented in this work will find further utility in identifying and characterizing relevant biological signal, yielding new understandings of complex genomic concepts hidden within this vast amount of data.

## Methods

### Data curation

TCR sequencing files were collected as raw tsv/csv formatted files (Supplementary Fig. 1) from the various sources cited within the manuscript. Sequencing files were parsed to take the amino acid sequence of the CDR3 after removing unproductive sequences. Clones with different nucleotide sequences but the same amino acid sequence were aggregated together under one amino acid sequence and their reads were summed to determine their relative abundance. Within the parsing code, we additionally specified to ignore sequences that used non-IUPAC letters (*,X,O) and removed sequences that were greater than 40 amino acids in length. For the purpose of the algorithm, the maximum length can be altered but we chose 40 as we did not expect any real sequences to be longer than this length.

### Data transformations

In order to allow a neural network to train from sequence data, we converted the amino acids to numbers between 0 and 19 representing the 20 possible amino acids. These were then one-hot encoded as to provide a categorical and discrete representation of the amino acids in numerical space. This process was applied prior to all networks being trained. For analyses where V/D/J gene usage, these genes were represented as categorical variables and one-hot encoded as inputs for the neural network.

### TCR featurization block

The core of all deep learning architectures is the TCR Featurization Block which takes the various sequence data for a given TCR and transforms it to a latent joint representation of all its inputs. For the α/β CDR3 sequences, we take variable length right-padded sequence data which has been encoded in one-hot representation and first apply an embedding layer which transforms this one-hot representation to a trainable continuous representation of dimensionality 64. This embedding layer learns features of each amino acid allowing the network to learn amino acids which may play similar roles in antigen-binding in the context of the TCR. Following this transformation, three convolutional layers are applied to the continuous representation of the CDR3 sequences. The kernel, stride sizes, and number of feature maps were (kernel: 5, stride: 1, feature maps: 32), (kernel: 3, stride: 3, feature maps: 64), (kernel: 3, stride: 3, feature maps: 128) respectively for the three layers. If the convolutional stack is being used within the VAE, the output of the final convolutional layer is flattened. If the convolutional stack is being used within either the supervised sequence classifier or repertoire classifier, the global max pooling operation is applied across the length of the sequence to provide the ability for the network to learn length-invariant motifs.

If V/D/J gene information is provided as an input to the network, this data are represented first as categorical variable with a one-hot encoding to the network. Once again, we apply a trainable embedding layer which transforms this one-hot representation to a continuous representation of dimensionality 48. This transformation produces the featurization of the V/D/J genes.

After all inputs to the network have been featurized, they are concatenated and this completes the TCR Featurization Block where a TCR is described by a vector of continuous variables that describe all of the possible CDR3 sequences and corresponding V/D/J gene usage. This TCR Featurization Block is used as the main building block for all networks described and used in the manuscript.

### Training VAE

In order to train the VAE, following creation of the computational graph as described in the manuscript and main figure, we applied an Adam Optimizer (learning rate = 0.001) to minimize a reconstruction loss and a variational loss. The reconstruction loss is the cross-entropy loss between the reconstructed sequence (S) and the one-hot encoded tensor of the input sequence (L) across the *i*th position in the sequence (1). The variational loss is the Kullback–Leibler (KL) divergence between the distributions of the latent variables and a unit Gaussian (2).

1 $$\,{R}_{\mathrm{{loss}}}=-\sum_{i}{L}_{i}\log({S}_{i})$$

2 $$\,{V}_{\mathrm{{loss}}}={D}_{\mathrm{{KL}}}(N(\mu (X),\sigma (X))| | N(0,1))$$

The variational loss serves as a regularizer to the network as it prevents overfitting of the network and direct memorization of sequence to latent space and allows for meaningful downstream clustering of the sequences in their latent representation^32,33^. The VAE was trained until convergence criteria were met. Features for all sequences were then extracted from the latent space and used for downstream analyses.

### Quantifying TCR distance

In order to quantify the distance between TCR sequences from the latent representations produced by the VAE, we computed a Euclidean distance in this space to measure the distance between any two TCR sequences. For the *K*-mer representation, we also used a Euclidean distance on the *K*-mer count vector to measure the distance between any two TCR sequences. To compute Hamming distance, we used the scipy.pdist function on the integer representation of the sequences. For the global sequence alignment-based distance, we computed a symmetric distance based as previously described by Sidhom et al. in the ImmunoMap algorithm. Global sequence alignment was computed with BioPython’s pairwise2.align.globalxx functionality^20^.

### Clustering antigen-specific TCR sequences

To assess the quality of the various featurization methods described in the study, we first applied an agglomerative clustering algorithm (scikit-learn) to the previously described TCR distances from the various VAE methods along with the Hamming, K-mer, and Global Sequence Alignment distance metrics. We varied the number of clusters for the algorithm from 5 to 100 clusters and measured the Variance Ratio Criterion (Calinski and Harabasz score) as well as the Adjusted Mutual Information across all the clustering solutions for all the described featurization methods^34,35^. By choosing these two metrics to quantify the robustness of the clustering solutions on the latent features, we first assessed the ratio of the within-cluster dispersion to the between-cluster dispersion as a measure for the “compactness” of the clustering solution via the Variance Ratio Criterion and then quantified using information theoretic principles to quantitate how much of the information about the antigen specificity was being captured by the clustering solution. Methods that provide a feature space optimal for applying clustering algorithms would have a high Variance Ratio Criterion as well as a high Adjusted Mutual Information. These metrics were applied to both the murine and human antigen-specific TCRs to compare the various featurization methods.

### Training K-nearest neighbor (KNN) algorithm on TCR sequences

In order to assess the quality of the various featurization methods describes in the study, we also applied a KNN on to the previously described TCR distances derived from the various VAE methods along with the Hamming, K-mer, and Global Sequence Alignment distance metrics. We employed a fivefold cross-validation strategy to split the data and then assessed performance on the left-out fold of the data. Furthermore, we varied the value of *K* in the KNN evenly from 1 to 500 in order to further assess the robustness of the featurization/KNN across a wide variety of *K* values. We were then able to do paired statistical analyses at each value of *K* to assess which featurization methods allowed for best downstream performance of the classifier to assign a TCR sequence to its correct cognate antigen.

### Training receptor classifier

In order to train the receptor classifier, we use the TCR Featurization Block as described previously to featurize the input data which can include any or all of α/β CDR3 and the corresponding V/D/J gene usage. The main difference in this featurization is that for the CDR3 sequences, we employ a global max pooling operation after the final convolutional layer to allow for translational invariance of motifs within the CDR3 sequence. The final feature space is directly sent to a classification layer where the number of final nodes is equivalent to the number of classes. In the case of a regression task where the receptor is being regressed to a continuous label, the feature space is sent to a single node. In the case of a classification task, the network is trained using an Adam Optimizer (learning rate = 0.001) to minimize the cross-entropy loss between the soft-maxed logits and the one-hot encoded representation of the discrete categorical outputs of the network. In the case of a regression task, the network is trained to minimize the mean squared error loss between the output of the final node in the network at the continuous label. Training was conducted by using 75% of the data for the training set, and 25% for validation and testing. The validation group of sequences was used to implement an early stopping algorithm.

### Training repertoire classifier

Designing an architecture for whole sample multi-instance classification presented unique challenges that were specific to the way TCR-Seq data is generated. Following featurization via the described TCR Featurization Block, we needed an architecture that could handle applying a label to a collection of these featurized sequences. In order to solve this multi-instance problem, we developed a multi-head attention mechanism that uses an adaptive activation function to make an assignment for each TCR sequence to a learned concept within the data. In order to design this activation function, we based it on the inverse square root unit (ISRU) function which is an algebraic form of the sigmoid function. While activation functions in neural networks are often fixed and have no trainable parameters (i.e. relu, sigmoid), we noted difficulty in training a sigmoid function to make an assignment between 0 and 1 due to the commonly cited problem of diminishing gradients with the use of sigmoid activation functions. By creating an adaptive ISRU function with a trainable *α* and *β* parameter (3), we found this improved the training of our network and allowed us to make a sequence-level assignment between 0 and 1 for each sequence to each learned concept in the model.

3 $$\,{\mathrm{{AISRU}}}=L+\left(\frac{H-L}{2}\right)\left(\frac{x}{{(a+{({x}^{2})}^{b})}^{\frac{1}{2b}}}\right)$$

$$\,L=0,\,H=1,\,a\, > \, 0,\,b\ge 1$$

This average of these assignments is taken over the sample to come up with what can be interpreted as the proportion of the repertoire that contains the learned concept. This vector of proportion features is then fed directly into the classification layer. The network is trained with an Adam Optimizer (learning rate = 0.001) to minimize the cross-entropy loss between the soft-maxed logits and the one-hot encoded representation of the discrete categorical outputs of the network.

### Motif identification

Neural networks are often treated as “black boxes” where their value is largely in their predictive performance and not in understanding how the neural network is accomplishing its task. However, in the area of the biological sciences, there is not only the desire to create predictive tools but use these tools to inform our own understanding of the mechanisms at play. This area of research is often termed as improving the “explainability” of neural networks. In biological sequence analytics such as DeepTCR, investigators want to be able to extract the features/motifs the neural network learned to accomplish its task. For the supervised learning architectures, we were able to identify motifs the network had learned by extracting the indices of where the kernels were activated following the global max pooling layer. The result of this operation is the network not only extracts the maximum value of a kernel over the length of the sequence but also deduces its position within the sequence. This can be then used to not only pick up which features are activated on a given sequence but where in the sequence this activation occurs, allowing us to identify the motifs that any given neuron in the net is learning.

### Statistical tests and machine learning models

All statistical tests applied to data were implemented with the scipy.stats module. Classical machine learning techniques and performance metrics were implemented with scikit-learn.

### Reporting summary

Further information on research design is available in the Nature Research Reporting Summary linked to this article.

## Supplementary information

Supplementary Information

Reporting Summary

## Data Availability

All data analyzed in this manuscript can be found at https://github.com/sidhomj/DeepTCR as well as in the original publications that generated the data^20–22,31^. The 10X Genomics dataset used to train the supervised sequence regression can be found at https://www.10xgenomics.com/resources/application-notes/a-new-way-of-exploring-immunity-linking-highly-multiplexed-antigen-recognition-to-immune-repertoire-and-phenotype/.

## Code availability

DeepTCR was written using Google’s TensorFlowTM deep learning library (https://github.com/tensorflow/tensorflow) and is available as a python package. Source code, comprehensive documentation, use-case tutorials, and all ancillary code (including all deep learning hyperparameters) to recreate all the figures in the text along with all data and results presented in this manuscript can be found at https://github.com/sidhomj/DeepTCR^55^. DeepTCR can either be installed directly from Github or from PyPI at https://pypi.org/project/DeepTCR/.
