# Author Correction: DeepTCR is a deep learning framework for revealing sequence concepts within T-cell repertoires

**DOI:** 10.1038/s41467-021-22667-2

**Published:** 2021-04-13

**Authors:** John-William Sidhom, H. Benjamin Larman, Drew M. Pardoll, Alexander S. Baras

**Affiliations:** 1 Bloomberg Kimmel Institute for Cancer Immunotherapy, Johns Hopkins University School of Medicine, Baltimore, MD USA; 2 The Sidney Kimmel Comprehensive Cancer Center, Johns Hopkins University School of Medicine, Baltimore, MD USA; 3 Department of Biomedical Engineering, Johns Hopkins University School of Medicine, Baltimore, MD USA; 4 Department of Pathology, Johns Hopkins University School of Medicine, Baltimore, MD USA

**Keywords:** Computational models, Functional clustering, Machine learning, Adaptive immunity, Immunogenetics

Correction to: *Nature Communications* 10.1038/s41467-021-21879-w, published online 11 March 2021.

The original version of this Article omitted the following funding information from the Acknowledgements: “The research was supported by the Bloomberg~Kimmel Institute for Cancer Immunotherapy, The Mark Foundation for Cancer Research, philanthropy of Susan Wojcicki and Dennis Troper in support of Computational Pathology at Johns Hopkins, the Johns Hopkins – Bristol Myers Squibb Immuno-Oncology Consortium, and the NIH Cancer Center Support Grant”.

Additionally, the original version of this Article omitted a reference to previous work in ‘Tickotsky N, Sagiv T, Prilusky J, Shifrut E, Friedman N. McPAS-TCR: a manually curated catalogue of pathology-associated T cell receptor sequences. *Bioinformatics*. **33**, 2924–2929 (2017)’. This has been added as reference 40 at the fourth sentence of the Results section ‘Supervised regression allows identification of antigen-specific TCRs in single-cell data’: ‘To independently validate whether these models trained on single-cell data learned salient antigen-specific features of the immune response, we collected experimentally validated CDR3 β sequences from the McPAS-TCR database^40^ for Flu-MP (influenza derived), BMLF1 (EBV derived), and MART1 (melanoma derived) epitopes and applied the respective models trained on the 10x Genomics dataset on these TCRs’. These errors have been corrected in the PDF and HTML versions of the Article.

