# Supplementary material for: DeepTCR is a deep learning framework for revealing sequence concepts within T-cell repertoires
Source: Nat Commun. 2021 Mar 11;12:1605. doi: 10.1038/s41467-021-21879-w (PMC7952906; doi:10.1038/s41467-021-21879-w)
Supplement: Supplementary file 1 — Supplementary Information [file 41467_2021_21879_MOESM1_ESM.pdf]

Supplementary Material

1. **Dataset Descriptions**
2. **Comparing clustering performance of Hamming Distance v. GLIPH**
3. **AUC Scores for K-Nearest Neighbors on Murine Antigens**
4. **Recall Scores for K-Nearest Neighbors on Murine Antigens**
5. **Precision Scores for K-Nearest Neighbors on Murine Antigens**
6. **F1 Scores for K-Nearest Neighbors on Murine Antigens**
7. **AUC Scores for K-Nearest Neighbors on Human Antigens**
8. **Recall Scores for K-Nearest Neighbors on Human Antigens**
9. **Precision Scores for K-Nearest Neighbors on Human Antigens**
10. **F1 Scores for K-Nearest Neighbors on Human Antigens**
11. **Assessing correlation between various distance metrics and length of the sequence**
12. **Benchmarking various machine learning methods to classify TCR sequences by their antigen-specificity**
13. **Classification Performance of Residue Sensitivity analysis to identify contact residues**
14. **Experimental Validation of Repertoire Classifier**
15. **GAG TW10 Multi-Class Sequence Classification Performance**
16. **Prediction values for Repertoire Classifier on GAG IW9 epitope family**

Dataset	Host	Pathology	Description
Glanville_2017	Human	Infectious Disease	T-cells were sorted and sequenced from peripheral blood mononuclear cells (PBMCs) from healthy donors for 7 Class-I specificities.
Sidhom_2017	Murine	Cancer	T-cells were expanded, sorted, and sequenced against tumor-associated antigens (SIY & TRP2) in the B16 cell line.
Dash_2017	Human & Murine	Infectious Disease	T-cells were sorted and sequenced from either mice or humans for 7 murine and 3 human Class-I specificities.
10x_Genomics	Human	Infectious Disease & Cancer	T-cells were high-throughput screened for a large number of human viral and tumor-associated antigens via published 10x Genomics single-cell pipeline.
Chan_2020	Human	Infectious Disease	T-cells were cultured with HIV-specific epitopes for 10 days prior undergoing TCR-Seq. Triplicates were conducted for each cognate epitope along with positive (CEF) and negative (No Peptide) controls.

Supplementary Figure 1. Dataset Descriptions. DeepTCR was piloted on sources of data that covered both human and mouse TCRs including samples taken from infectious disease settings and cancer pathology.

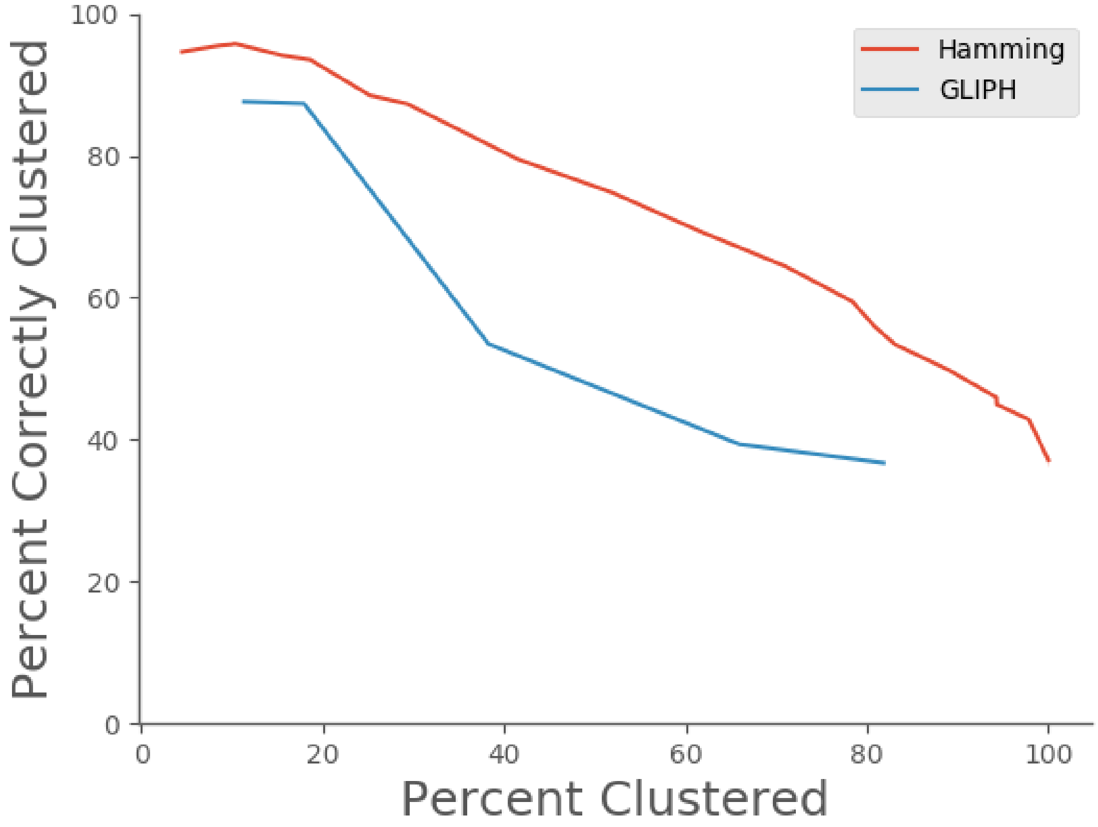

Supplementary Figure 2. Comparing clustering performance of Hamming Distance v. GLIPH. In order to assess the performance of a simple Hamming distance vs the state-of-the-art TCR-Seq clustering algorithm, we applied a Hamming distance followed by hierarchical clustering following Ward linkage to the *Glanville* dataset of 2066 TCR sequences specific for 7 antigens as well as ran the GLIPH clustering algorithm. We then assessed the clustering accuracy (as per methods used by *Glanville et. al.*). This entailed only including clusters with at least 3 sequences and making cluster assignments based on the majority of members within a cluster. If there was no majority, no cluster assignment was made. For clusters with assignments, sequences within the cluster that shared the cluster assignment labeled were counted as being clustered correctly.

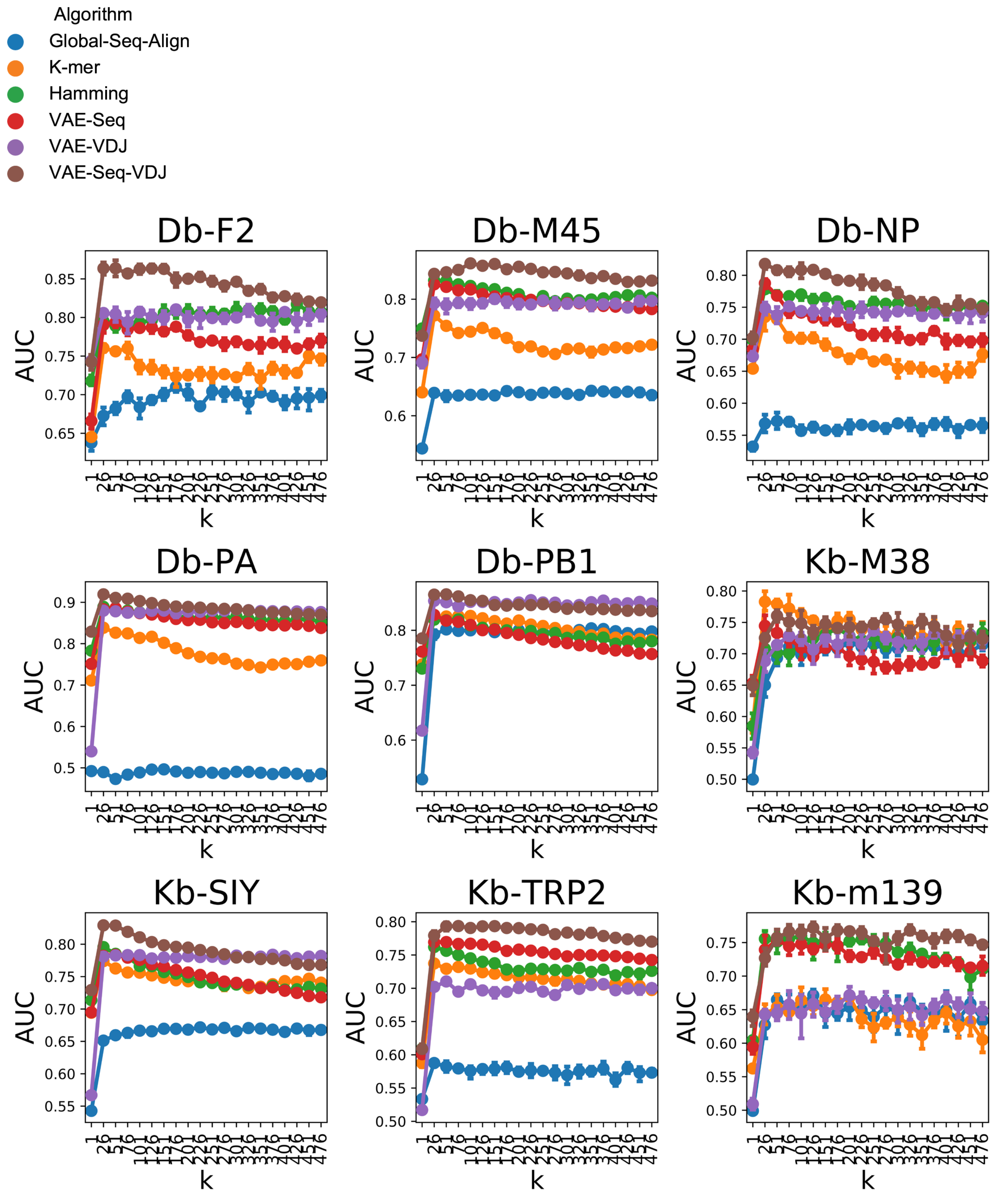

Supplementary Figure 3. AUC Scores for K-Nearest Neighbors on Murine Antigens. A K-Nearest Neighbors algorithm was applied using a 5-Fold Cross-Validation across various values for k. AUC performance was assessed across all methods. For each k, n=5 cross-validations, where mean value is shown along with the 95% confidence interval.

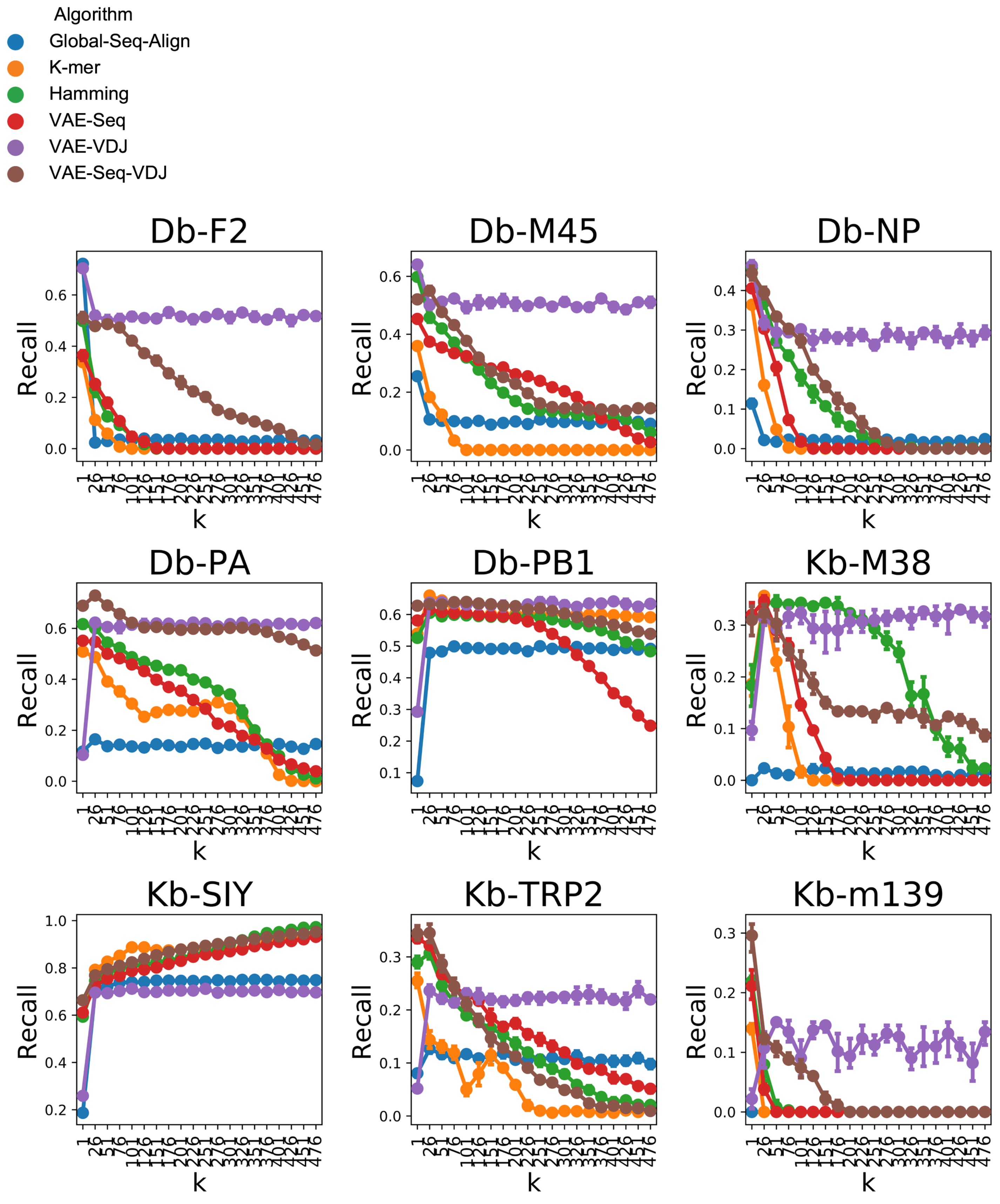

Supplementary Figure 4. Recall Scores for K-Nearest Neighbors on Murine Antigens. A K-Nearest Neighbors algorithm was applied using a 5-Fold Cross-Validation across various values for k. Recall performance was assessed across all methods. For each k, n=5 cross-validations, where mean value is shown along with the 95% confidence interval.

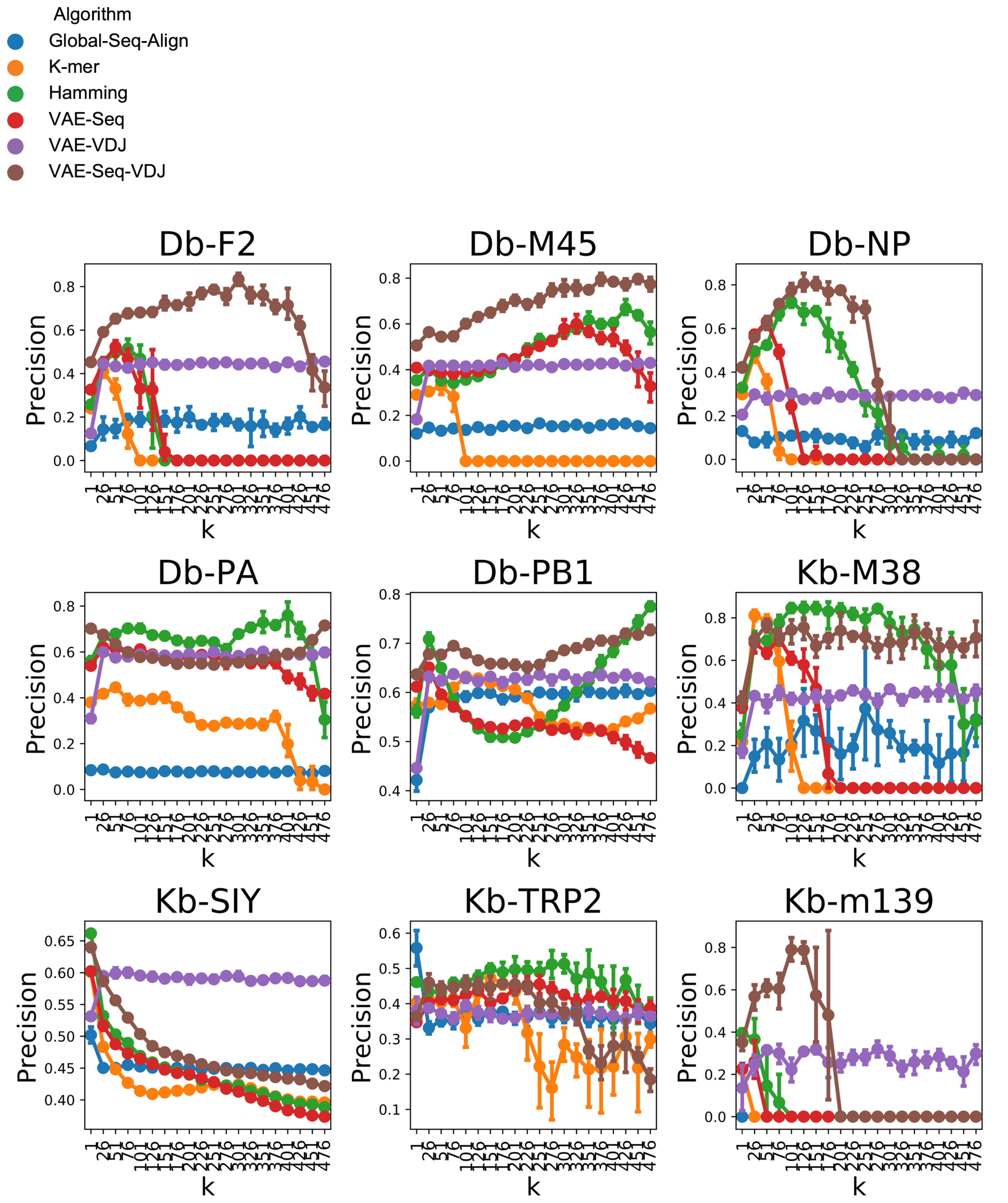

Supplementary Figure 5. Precision Scores for K-Nearest Neighbors on Murine Antigens. A K-Nearest Neighbors algorithm was applied using a 5-Fold Cross-Validation across various values for k. Precision performance was assessed across all methods. For each k, n=5 cross-validations, where mean value is shown along with the 95% confidence interval.

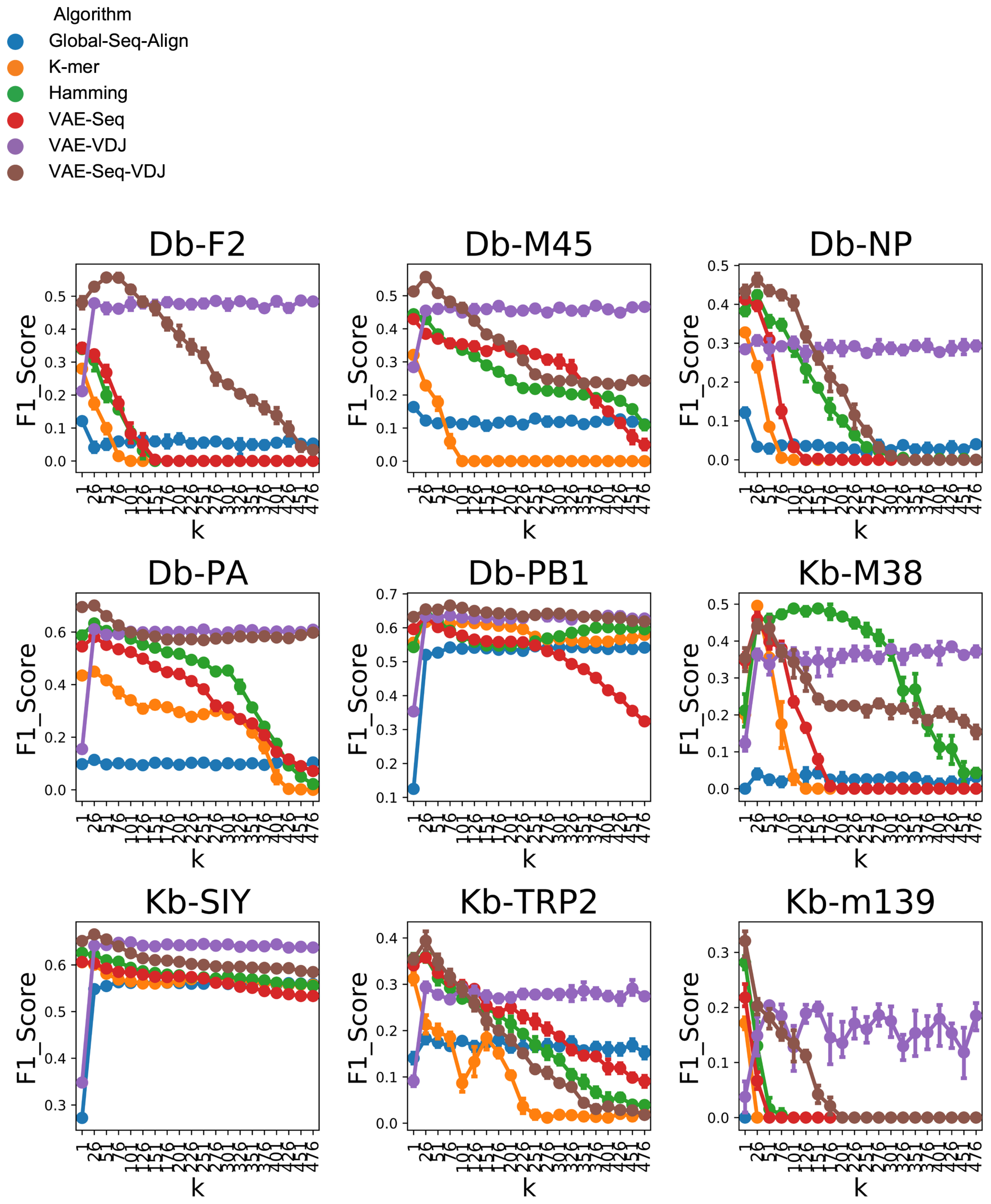

Supplementary Figure 6. F1 Scores for K-Nearest Neighbors on Murine Antigens. A K-Nearest Neighbors algorithm was applied using a 5-Fold Cross-Validation across various values for k. F1 Score was assessed across all methods. For each k, n=5 cross-validations, where mean value is shown along with the 95% confidence interval.

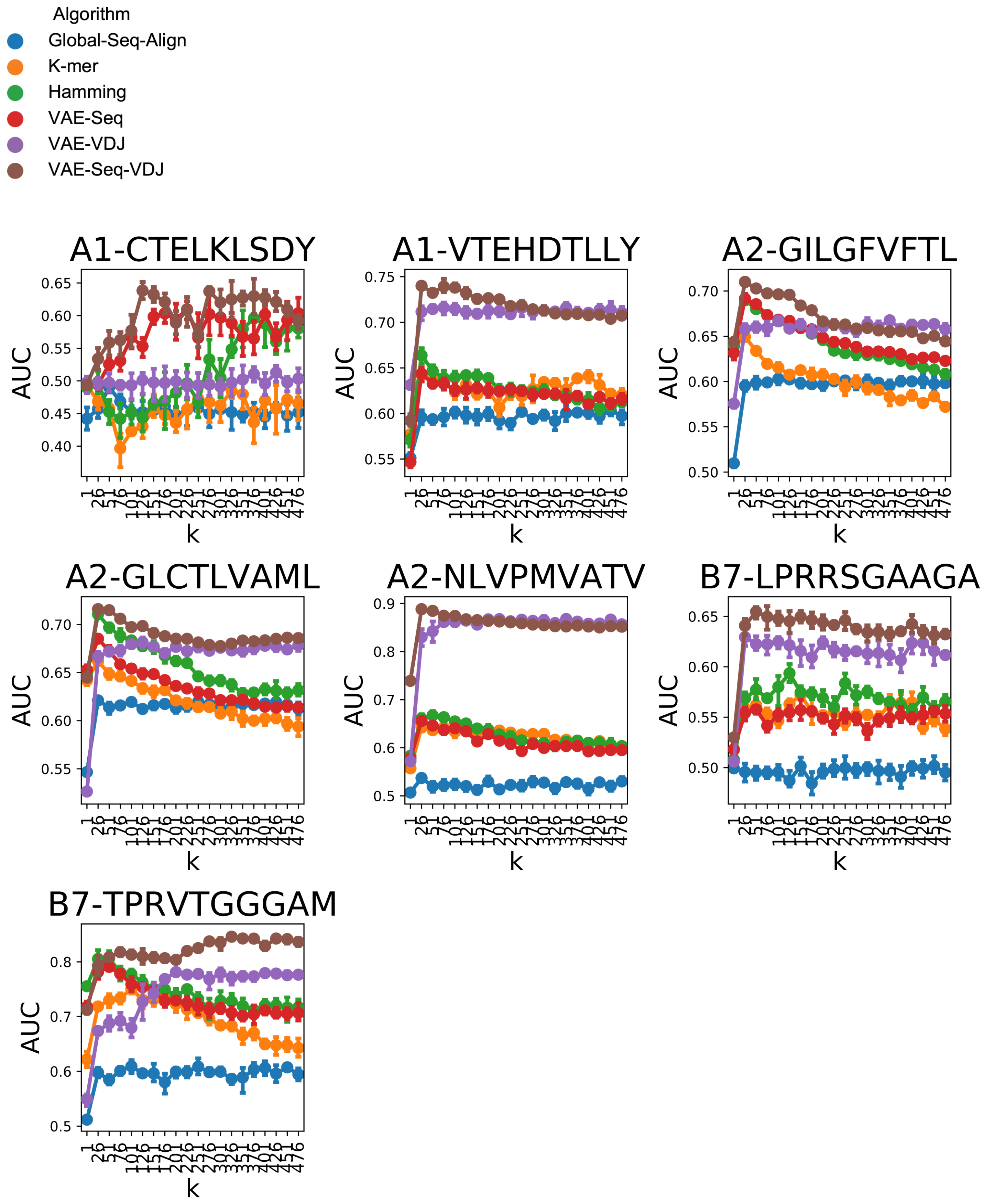

Supplementary Figure 7. AUC Scores for K-Nearest Neighbors on Human Antigens. A K-Nearest Neighbors algorithm was applied using a 5-Fold Cross-Validation across various values for k. AUC performance was assessed across all methods. For each k, n=5 cross-validations, where mean value is shown along with the 95% confidence interval.

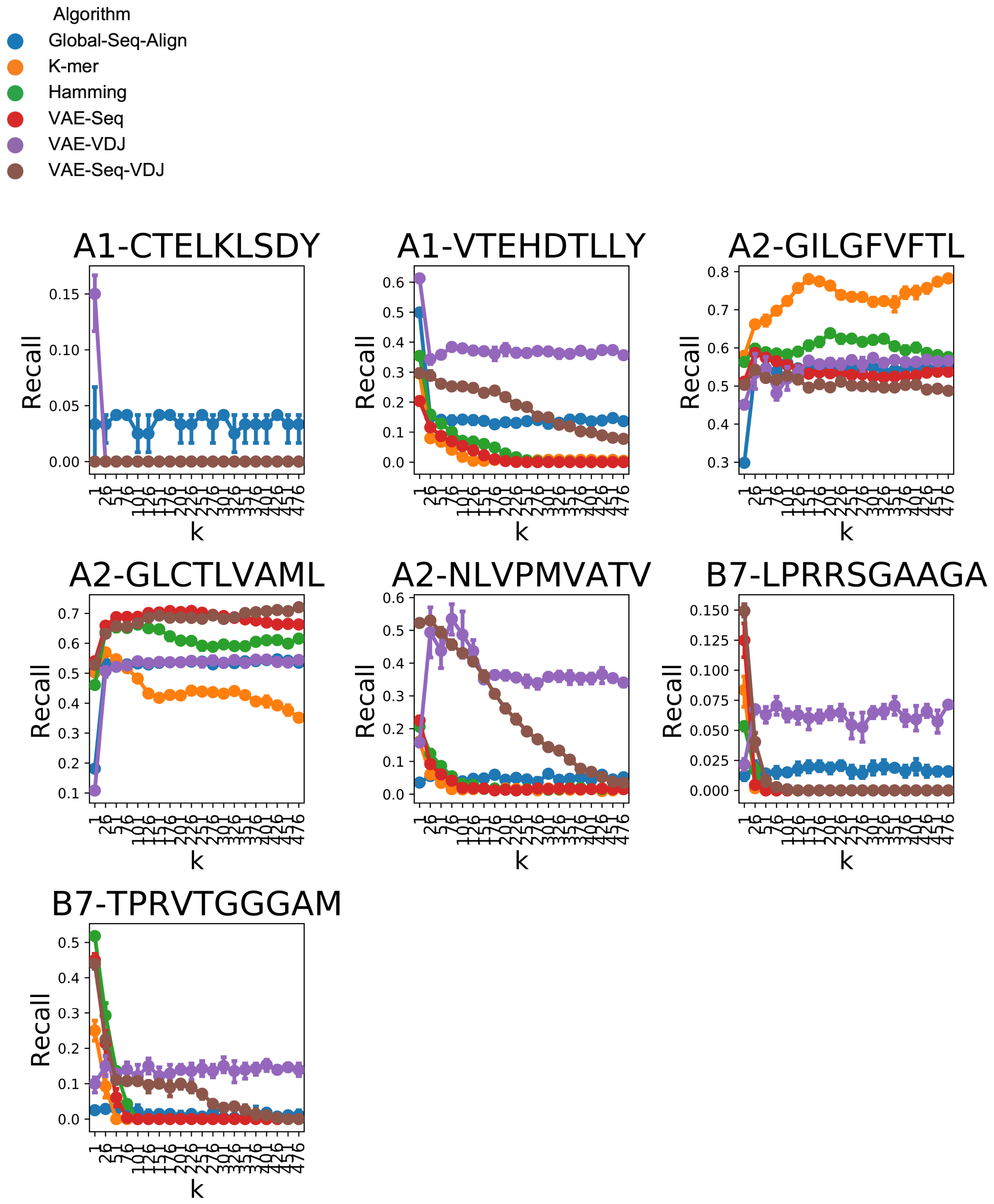

Supplementary Figure 8. Recall Scores for K-Nearest Neighbors on Human Antigens. A K-Nearest Neighbors algorithm was applied using a 5-Fold Cross-Validation across various values for k. Recall performance was assessed across all methods. For each k, n=5 cross-validations, where mean value is shown along with the 95% confidence interval.

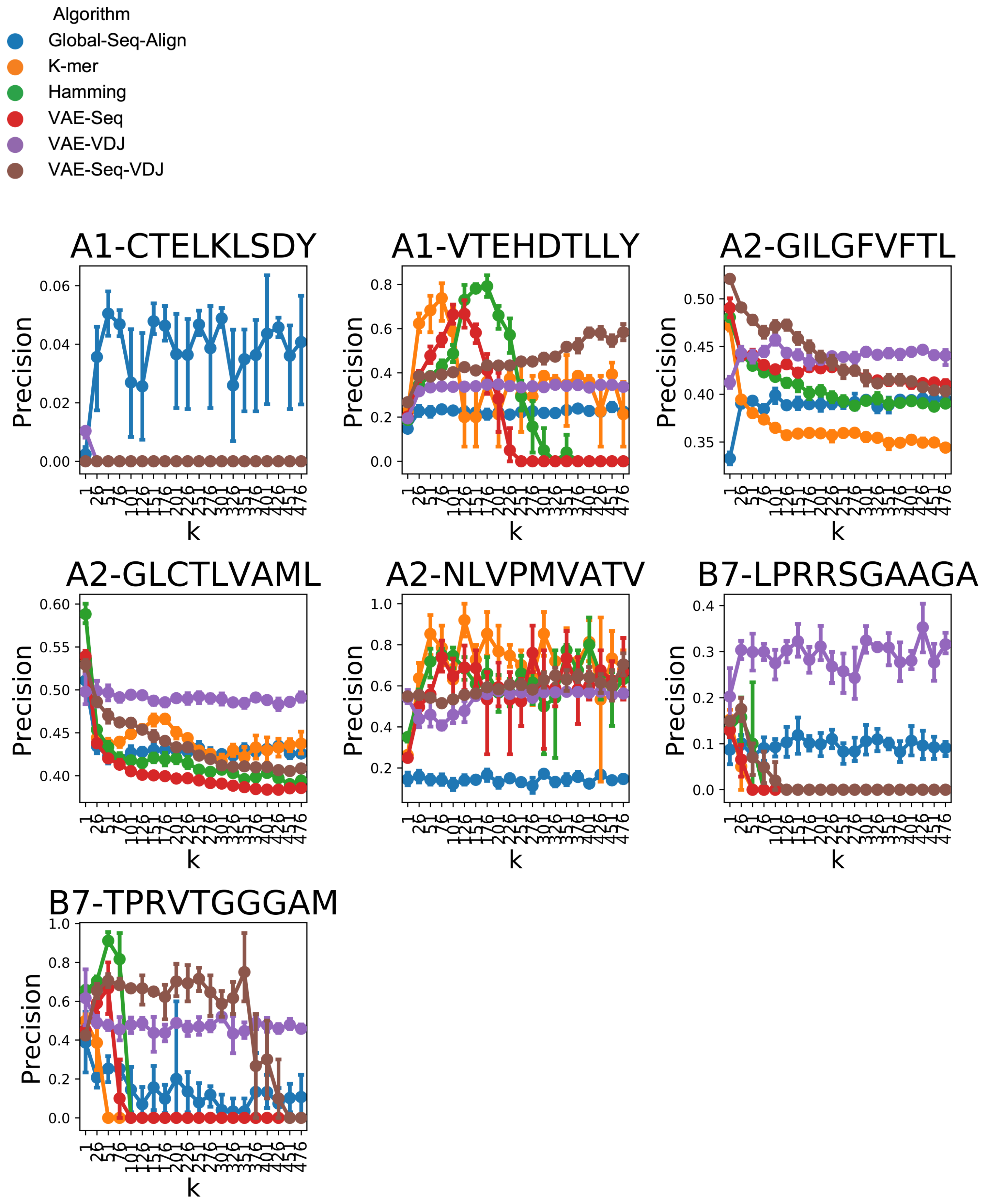

Supplementary Figure 9. Precision Scores for K-Nearest Neighbors on Human Antigens. A K-Nearest Neighbors algorithm was applied using a 5-Fold Cross-Validation across various values for k. Precision performance was assessed across all methods. For each k, n=5 cross-validations, where mean value is shown along with the 95% confidence interval.

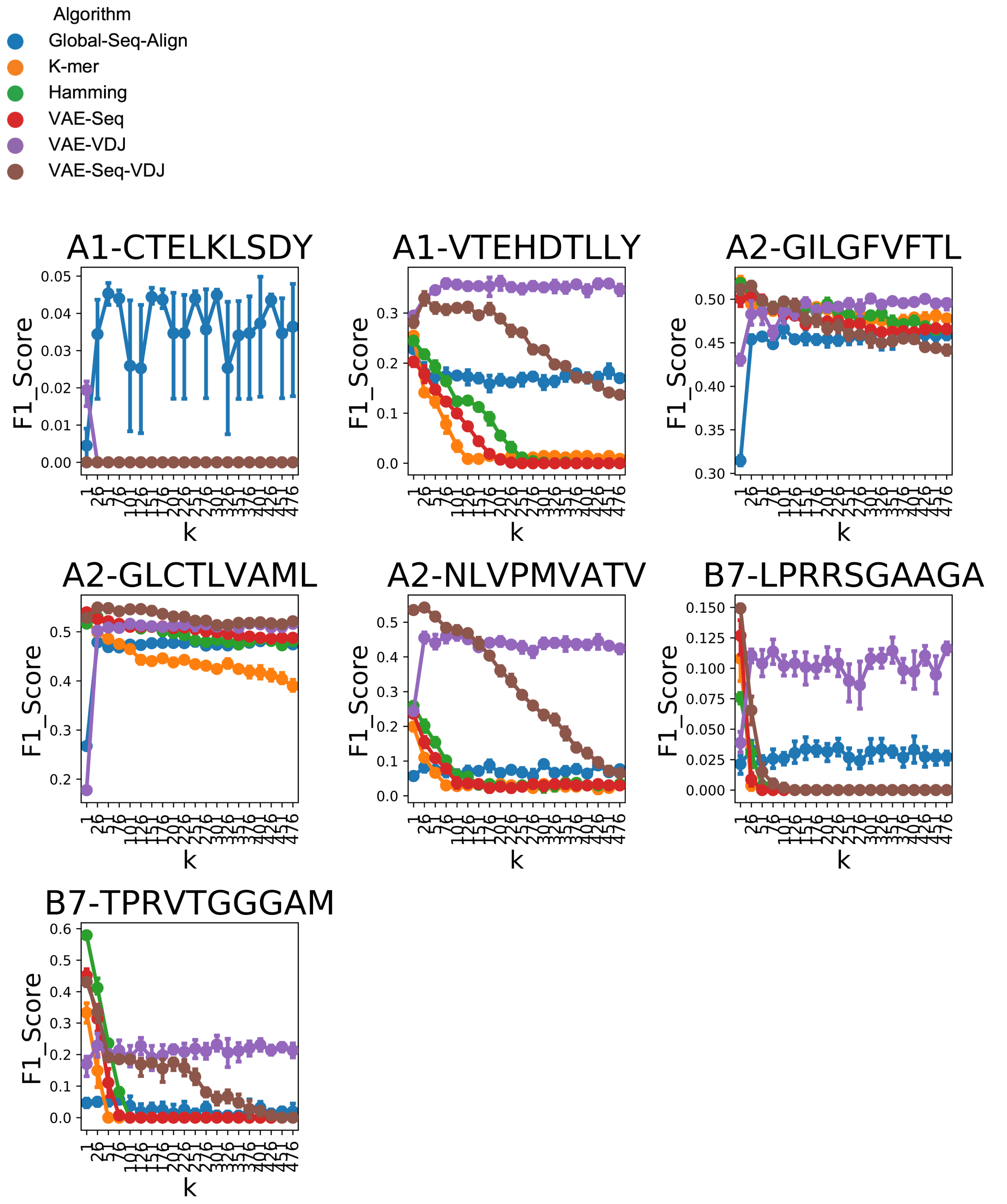

Supplementary Figure 10. F1 Scores for K-Nearest Neighbors on Human Antigens. A K-Nearest Neighbors algorithm was applied using a 5-Fold Cross-Validation across various values for k. F1 Score was assessed across all methods. For each k, n=5 cross-validations, where mean value is shown along with the 95% confidence interval.

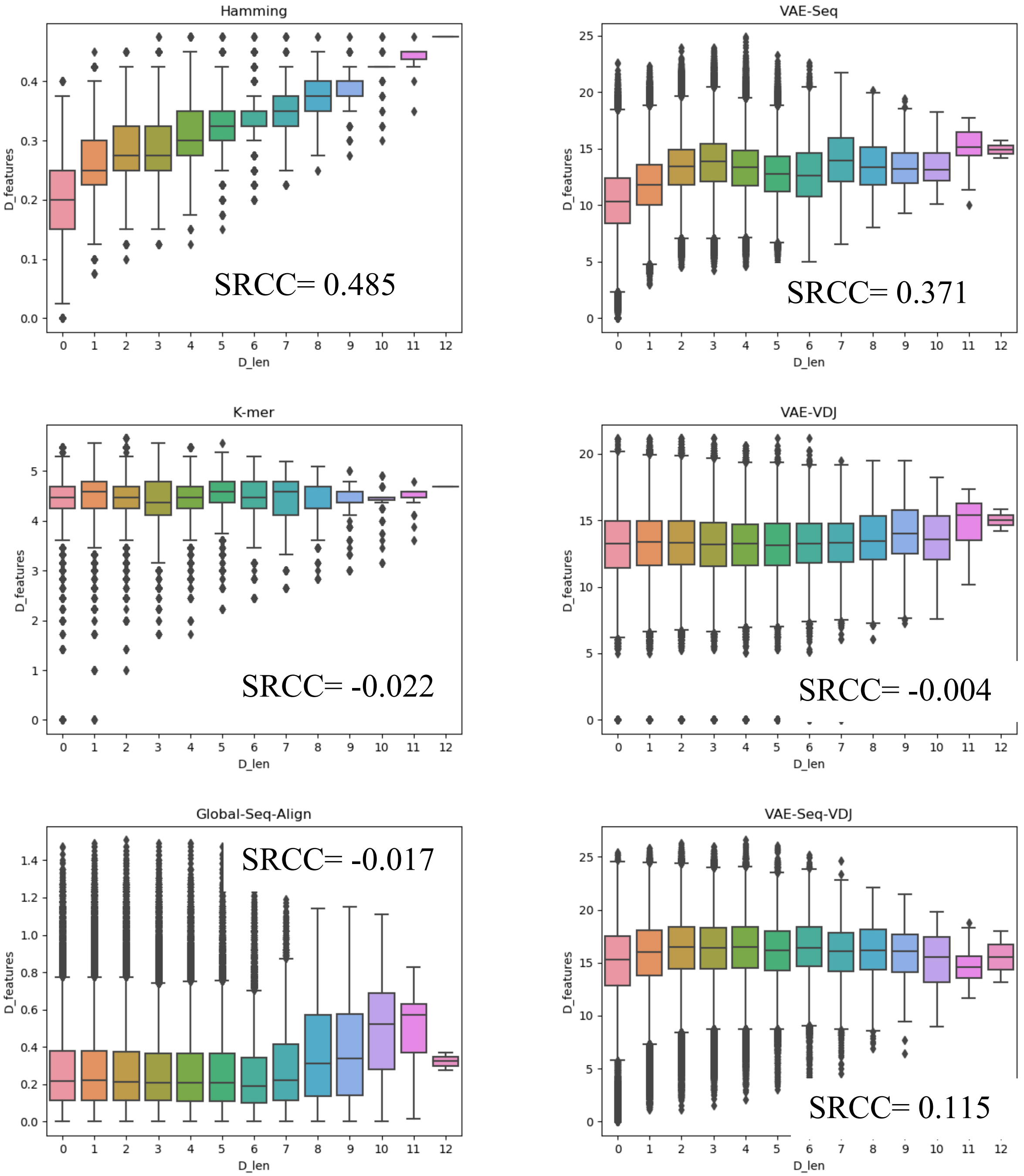

Supplementary Figure 11. Assessing correlation between various distance metrics and length of the sequence. In order to assess the extent by which these various methods used to quantify the distance between sequences was driven by sequence length, we determined the Spearman's Rank Correlation Coefficient (SRCC) between the distances of a given pair of sequences and how different in length they were. Boxplots are shown to compare distance by sequence length (x-axis) by distance of various methods (y-axis), where median and interquartile range (IQR) are shown for each length sequence and outliers are defined as Q1 - 1.5*IQR or Q3+1.5*IQR.

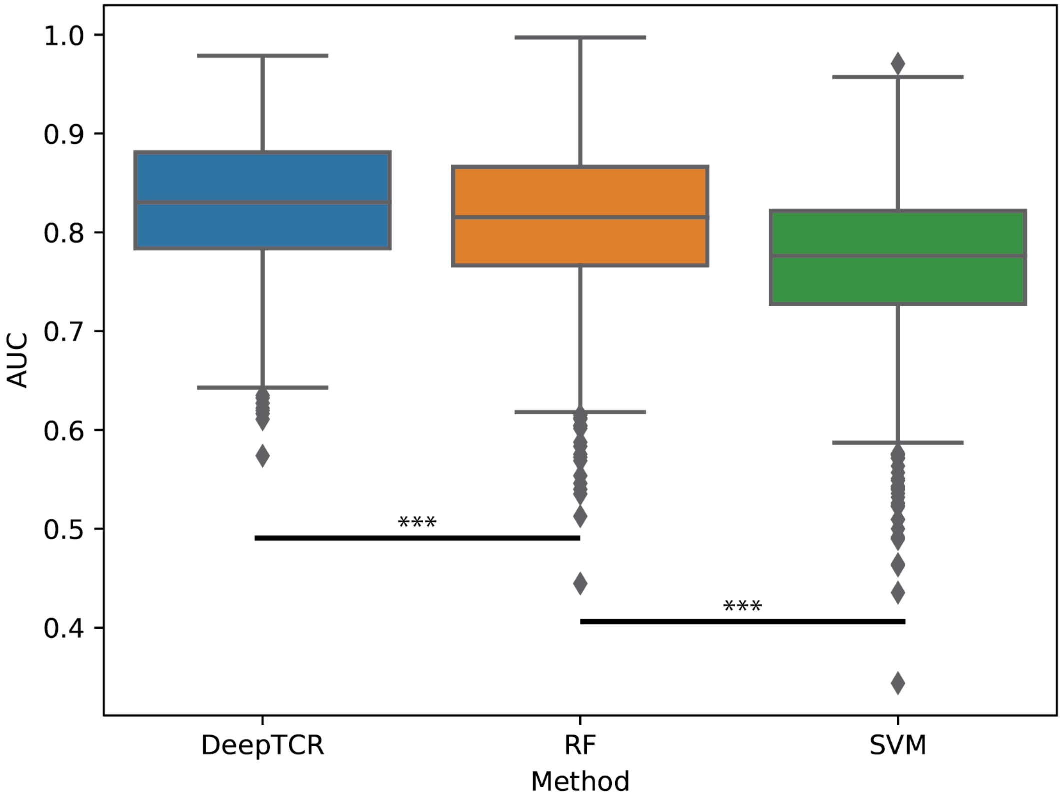

Supplementary Figure 12. Benchmarking various machine learning methods to classify TCR sequences by their antigen-specificity. In order to compare the performance of various machine learning approaches to classify TCR sequences by their antigen-specificity, we used the TCR sequences for the 9 murine antigens collected to benchmark DeepTCR's deep learning classifier vs a classical Support Vector Machine (SVM) and Random Forrest (RF) classifier. Only the beta cdr3 sequence was provided to all 3 machine learning algorithms to test the ability of the classifiers to predict antigen-specificity from cdr3 motifs. 100 iterations of a 5-fold cross-validation were completed and AUC's were measured for each of the various methods to assess classification performance. Average AUC values for DeepTCR's deep learning sequence classifier, the Random Forrest, and SVM were 0.830, 0.810, and 0.769 respectively. (Two-tailed independent t-test, * : $p < 0.05$, ** : $p < 0.01$, *** : $p < 0.001$). Boxplots are shown to compare performance by each method where median and interquartile range (IQR) are shown for each length sequence and outliers are defined as Q1 - 1.5*IQR or Q3+1.5*IQR.

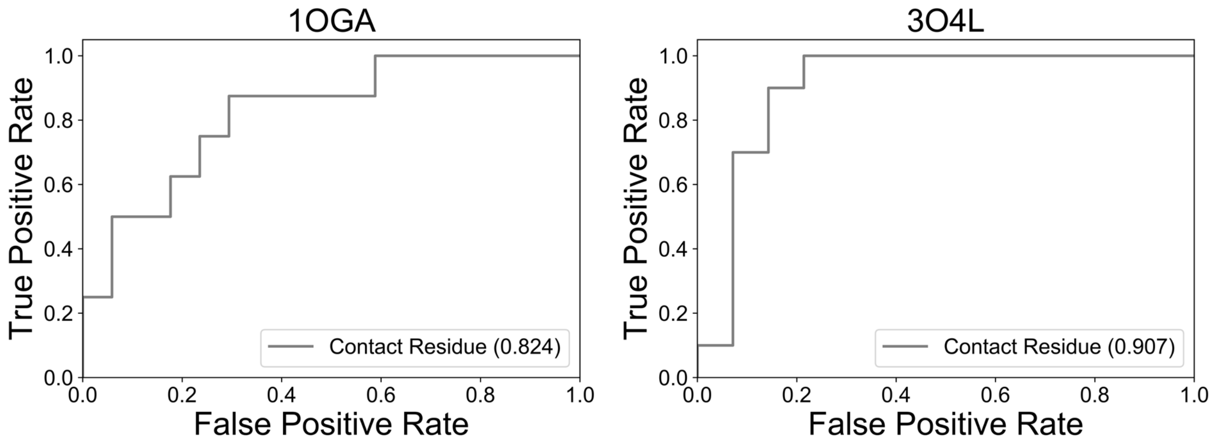

Supplementary Figure 13. Classification Performance of Residue Sensitivity analysis to identify contact residues. Following creation of Residue Sensitivity Logos for Flu-MP (1OGA) and BMLF1 (3O4L) TCRs, the range of perturbation values at every position (height of the logo) was used as a predictor of being a contact residue. ROC curves were constructed, and AUC's were computed to assess the predictive power.

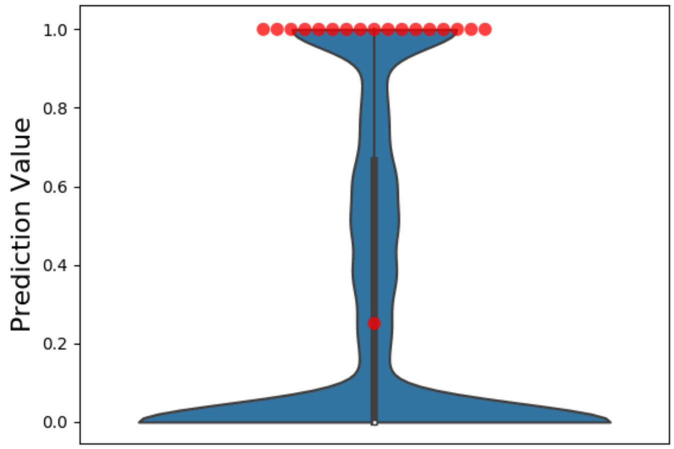

TCR	Peptide	Prediction Value
CAISLMGTEAFF	HTQGYFPDW	1.000
CAISLMGTEAFF	NTQGYFPDW	1.000
CASSLDLRTFTYEQYF	KAALDLSHF	1.000
CASSLDLRTFTYEQYF	KSALDLSHF	1.000
CASSLDPGANTEAFF	TSTLQEQIGW	1.000
CASSLDPGANTEAFF	TSTLAEQMAW	1.000
CASSLERVGYNEQFF	KAALDLSHF	1.000
CASSLLAGGSLDEQFF	KAAVDLSHF	1.000
CASSLLAGGSLDEQFF	KAALDLSHF	1.000
CASSLLAGGSLDEQFF	KSALDLSHF	1.000
CASSPGVGNTEAFF	TSTLQEQIGW	1.000
CASSPGVGNTEAFF	TSTLAEQIAW	1.000
CASSPGVGNTEAFF	TSTLSEQVAW	0.251
CASSPGVGNTEAFF	TSTLAEQMAW	1.000
CASSPRQAGLVTQYF	TSTLAEQMAW	1.000
CASSPRWGDAGELFF	KAALDLSHF	1.000
CASSPRWGDAGELFF	KSALDLSHF	1.000
CAWETGVRDGYTF	KAALDLSHF	1.000

Supplementary Figure 14. Experimental Validation of Repertoire Classifier. Distributions of TCR-level predictions from 18 originally reported experimentally validated TCR-peptide pairs (red circles) along with all TCR-peptide pairs (blue background distribution) are shown via violin plot (left) with corresponding DeepTCR prediction values for validated pairs in table (right).

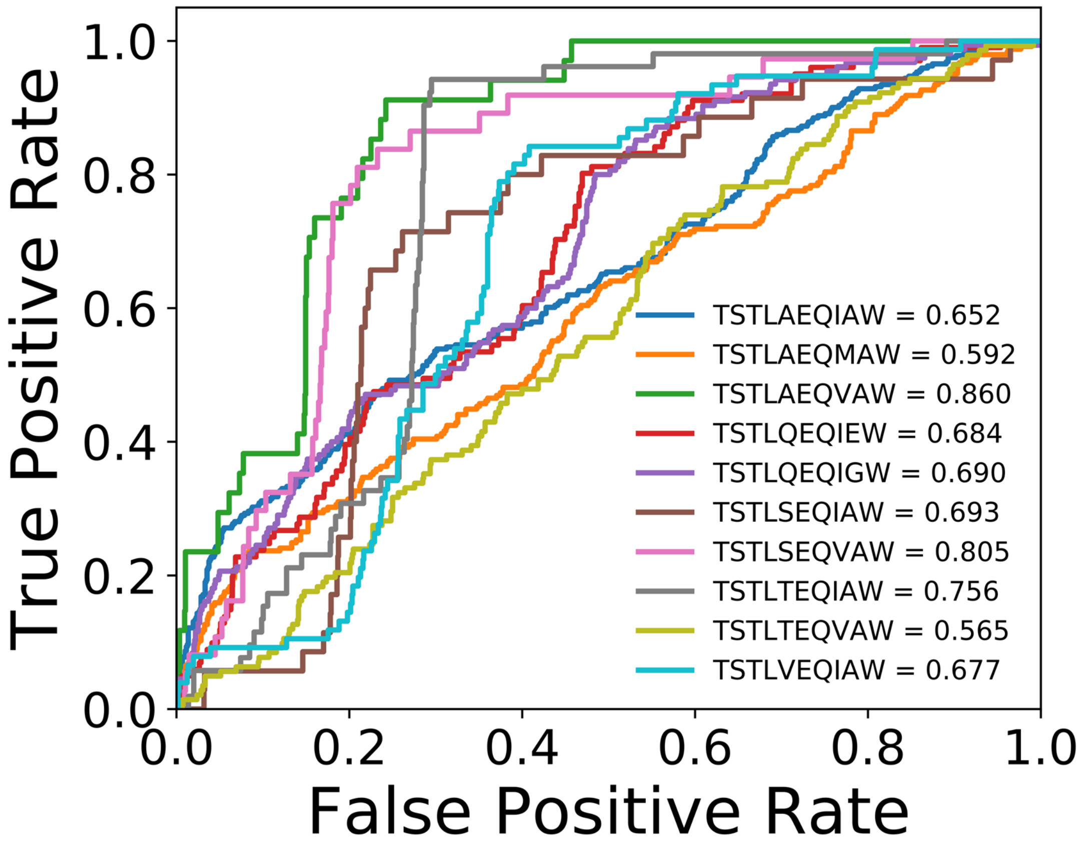

Supplementary Figure 15. GAG TW10 Multi-Class Sequence Classification Performance. Following initial screen to select antigen-specific immune expansion, we collected the positive predicted TCR sequences (prob > 0.99) from the autologous GAG TW10 variants and trained a TCR sequence classifier to predict based on TCR sequence which GAG TW10 variant the TCR recognized. ROC curves are shown for all variants in one v. all fashion.

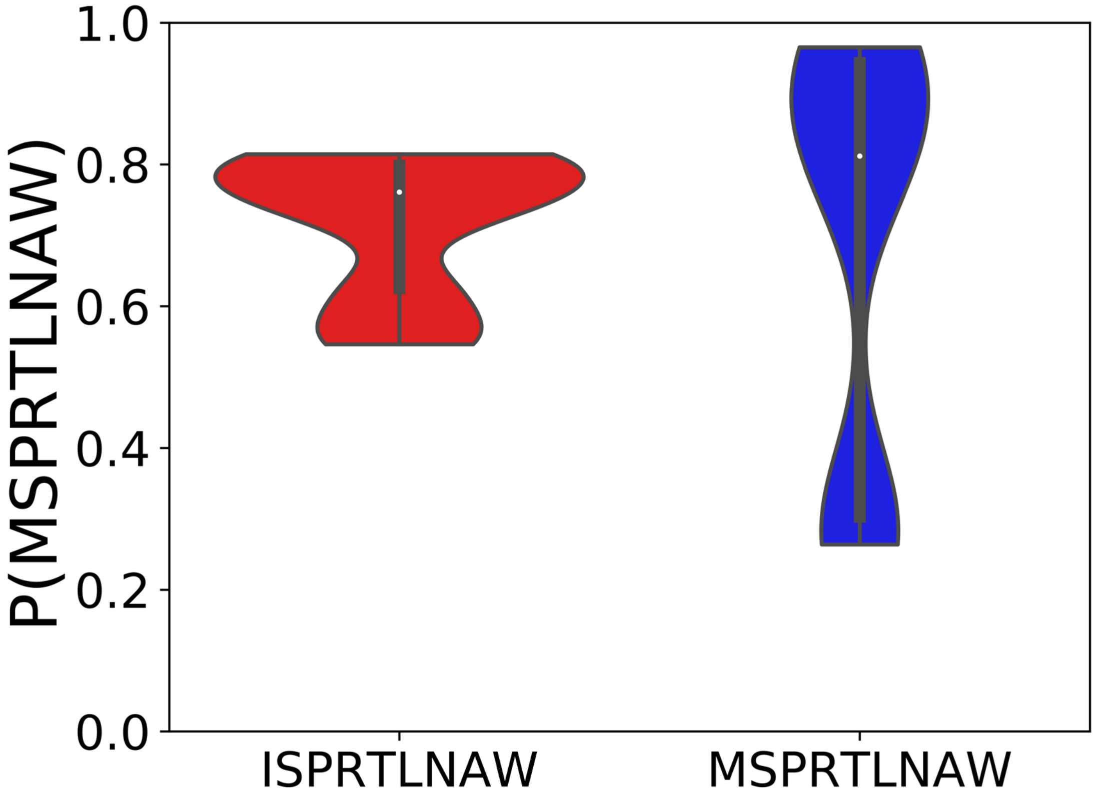

Supplementary Figure 16. Prediction values for Repertoire Classifier on GAG IW9 epitope family. The DeepTCR Repertoire Classifier was applied to the autologous variants from the GAG IW9 epitope family for ES8. Prediction values following 100 Monte-Carlo simulations are shown demonstrating inability for classifier to differentiate between the consensus and escape variant repertoires.
